# Supplementary material for: A novel yeast hybrid modeling framework integrating Boolean and enzyme-constrained networks enables exploration of the interplay between signaling and metabolism
Source: PLoS Comput Biol. 2021 Apr 9;17(4):e1008891. doi: 10.1371/journal.pcbi.1008891 (PMC8059808; doi:10.1371/journal.pcbi.1008891)
Supplement: S1 Text — Includes a detailed description of mechanisms reflected in the Boolean model of nutrient signaling as well as open questions of dynamics and model gaps. (DOCX) [file pcbi.1008891.s001.docx]

Supporting information on the Boolean layer

A novel yeast hybrid modeling framework integrating Boolean and enzyme-constrained networks enables exploration of the interplay between signaling and metabolism

Linnea Österberg^1,2,3^, Iván Domenzain ^3,4^, Julia Münch^1,2^, Jens Nielsen^3,4,5^, Stefan Hohmann ^3^, Marija Cvijovic^1,2*^

^1^ Department of Mathematical Sciences, University of Gothenburg, Gothenburg, Sweden

^2^ Department of Mathematical Sciences, Chalmers University of Technology, Gothenburg, Sweden

^3^ Department of Biology and Biological Engineering, Chalmers University of Technology, Gothenburg, Sweden

^4^ Novo Nordisk Foundation Center for Biosustainability, Chalmers University of Technology, SE41296 Gothenburg, Sweden

^5^ BioInnovation Institute, Ole Maaløes Vej 3, DK2200 Copenhagen, Denmark

*** Correspondence:**

Marija Cvijovic
[marija.cvijovic@chalmers.se](mailto:marija.cvijovic@chalmers.se)

# A detailed description of mechanisms reflected in the Boolean model of nutrient signaling

## SNF1

Snf1 activation upon glucose depletion is associated with increased phosphorylation of Thr210 mediated by the upstream kinases Tos3, Sak1 and Elm1 which are partially redundant, this activation appears to be constitutively (Hong et al., 2003; Sutherland et al., 2003). The return to the inactive state has been attributed to the Reg1-Glc7 complex where studies show that Reg1 interacts with Snf1 (Ludin et al., 1998) and targets Glc7 to the complex(Sanz et al., 2000). The model suggests that Reg1-Glc7 binds to Snf1p, mainly relevant in low glucose conditions, and Snf1p phosphorylates Reg1, Glc7 dephosphorylates Reg1. Hxk2 is then either 1) promoting binding of Reg1 to Snf1, 2) promoting phosphorylation of Reg1 or 3) interfering with dephosphorylation by Glc7. In response to high glucose, Reg1-Glc7 is dephosphorylating and thus releasing Snf1 from the complex. The dephosphorylation of Reg1 appears to increase the efficiency of Glc7 dephosphorylation as well as allowing Reg1 to be released from SNF1(Sanz et al., 2000). This complex form of regulation is a highly adaptable system with a fast response. In our model, this is implemented in a way that when glucose is absent Snf1 is phosphorylated by the upstream kinases. The SNF1 complex phosphorylates Reg1-Glc7. To account for the involvement of Hxk2 in our model we chose to implement mechanism number 2) where Hxk2p can phosphorylate Reg1 (Fernández-García et al., 2012). Phosphorylated Reg1 obstructs the activation of Snf1 and activates Glc7. In this low glucose state, SNF1 is active, Reg1 and Hxk2 are phosphorylated and Glc7 is active. When glucose is added, Hxk2 is unphosphorylated and a “high glucose signal”, in our model mediated through the PKA pathway (Barrett et al., 2012; Castermans et al., 2012) allows Glc7 to dephosphorylate Snf1. When Snf1 and Hxk2 are no longer phosphorylated Reg1 gets dephosphorylated by Glc7 and also Glc7 becomes inactive. In this high glucose condition, Snf1 and Reg1 are unphosphorylated and Glc7 is inactive. SNF1-mediates phosphorylation of the transcriptional factors Mig1, Cat8, Sip4 and Adr1 as well as directly phosphorylates and inactivates ACC1(Woods et al., 1994). Mig1 is a repressor that is active in high glucose conditions and represses genes used for alternative carbon sources, mainly SUC, MAL and GAL genes (Broach, 2012; Santangelo, 2006; Schüller, 2003; Westholm et al., 2008). In absence of glucose, Cat8 and Sip4 are activating the transcription of genes regulated by carbon source-responsive elements (CSRE) such as FBP1, PCK1 and ICL1 (Broach, 2012; Leverentz & Reece, 2006; MacPherson et al., 2006; Turcotte et al., 2010). Adr1 induces genes involved in the use of alternative carbon sources such as ADH1, ACS1 and GUT1 as well as peroxisome biogenesis and fatty acid utilization such as POX1 and PXA1(Broach, 2012; Kacherovsky et al., 2008; Smith et al., 2011; Soontorngun et al., 2012; Turcotte et al., 2010) It has been shown that PKA can inactivate the Adr1(Cherry et al., 1989).

## PKA pathway

The protein kinase A (PKA)/cAMP pathway mainly represses genes involved in stress tolerance and post diauxic growth when glucose is available. This means that properties associated with slow, reparative growth and stationary phase are negatively regulated by glucose (Conrad et al., 2014). Intra- and extracellular glucose sensing is carried out by two distinct G-protein systems, namely the Ras pathway and the Gpr1/Gpa2 pathway (Rolland et al., 2000). Ras proteins are small monomeric GTP-binding proteins that are regulated through a cycle of GDP/GTP exchange and GTP hydrolysis. This process is regulated by Cdc25 that triggers the exchange from GDP to GTP on the one hand (Broek et al., 1987; Jones et al., 1991; Robinson et al., 1987) and by Ira1 and Ira2 that stimulate GTP hydrolysis on the other hand(K Tanaka et al., 1989, 1990; Kazuma Tanaka et al., 1990). Sensing of extracellular glucose occurs via the G-protein coupled receptor (GPCR) Gpr1 that interacts with Gpa1. Glucose availability causes a Gpr1-mediated nucleotide exchange in Gpa2 from GDP to GTP yielding its activation (Colombo et al., 1998; Kraakman et al., 1999). Activated Gpa2 as well as/together with activated Ras can stimulate cellular cAMP production via the adenylate cyclase (AC) (Kataoka et al., 1985; Rolland et al., 2000; Takashi Toda et al., 1985). GPCRs require Ras activation to activate AC which requires activity in the upper metabolism such as activity in hexose kinases (Rolland et al., 2000). It has been shown that the accumulation of F16BP is coupled to Ras activation (K. Peeters et al., 2017). RAS mutants imitate the AC mutants and the lethality of RAS deletion can be alleviated by *bcy1* mutant cells in the same fashion as AC mutants (Takashi Toda et al., 1985). In the model, this is implemented so that Cdc25 is activated by F1,6BP which is present when glucose is present, and Ira is active when no glucose is present. In this model, Ras can activate AC but Gpa2 also requires active Ras to activate AC. AC is deactivated by crosstalk with the SNF1 pathway (Nicastro et al., 2015). The protein kinase A (PKA) is a heterotetrameric protein complex consisting of two catalytic (Tpk1-3) and two regulatory (Bcy1) subunits(Matsumoto et al., 1982; T Toda et al., 1987; Takashi Toda et al., 1987). The binding of cAMP to the Bcy1 subunits causes the complex to dissociate, thus releasing the blockade of Tpk1-3 kinase activity (Conrad et al., 2014). In contrast, the kelch repeat proteins Krh1 and Krh2 stimulate the association of catalytic and regulatory subunits resulting in an increased amount of cAMP required for PKA activation. However, it was shown that active Gpa2 inhibits Krh activity (T. Peeters et al., 2006). Upon the numerous PKA targets are the phosphodiesterases Pde1 and Pde2 which conciliate a negative feedback mechanism on PKA itself by degrading cAMP (Hu et al., 2010; Ma et al., 1999; Nikawa et al., 1987; Sass et al., 1986). This is implemented in the model so that the PKA complex is defined as the catalytic subunits and the regulatory subunit is required when the PKA complex is inactive. To activate PKA, the Krh proteins have to be inactive. In the model only phosphorylated Pde breaks down cAMP. PKA also inactivates the Rim15 protein kinase by phosphorylation which is therefore not able to activate the transcription factors Msn2, Msn4 and Gis1 (Swinnen et al., 2006). This can also be achieved through crosstalk with the TOR pathway (Wanke et al., 2008). In an active state, the former two induce expression of genes containing a stress response element (STRE) in their promoter whereas the latter induces transcription of genes comprising a post diauxic shift (PDS) element in their promoter (Martínez-Pastor et al., 1996; Pedruzzi et al., 2000). PKA also directly phosphorylates cytosolic enzymes such as trehalase (Schepers et al., 2012), phosphofructokinase 2 (Dihazi et al., 2003), pyruvate kinase (Portela et al., 2002) and fructose-1,6-bisphosphatase (Rittenhouse et al., 1987).

## TOR pathway

The target of Rapamycin (Tor) kinase complex 1 (TORC1) is not directly involved in glucose sensing; however, glucose availability has been shown to highly influence the activity of TORC downstream targets (Hughes Hallett et al., 2014). The strongly conserved TORC1 pathway plays a crucial role in promoting anabolic processes and cell growth in response to nitrogen availability which is probably sensed as the level of intracellular amino acids (Broach, 2012). TORC1 comprises either Tor1 or Tor2 kinase in association with Kog1, Lst8 and Tco89 (Reinke et al., 2004) and its activity is regulated by the EGO complex consisting of Ego1, Ego2, Gtr1 and Gtr2 (Dubouloz et al., 2005). Multiple complex nitrogen-sensing mechanisms (Bar-Peled et al., 2013; Binda et al., 2009; Bonfils et al., 2012) lead to the physical interaction of EGO with TORC1 resulting in activation of the latter under nitrogen-rich conditions (Binda et al., 2009). Active TORC1 then induces several signaling branches - the Sch9 branch, the Tap42-PPase branch as well as the activation of further transcription factors such as Sfp1 (Marion et al., 2004; Urban et al., 2007; Yan et al., 2006). TORC1 phosphorylates Sch9(Urban et al., 2007) which then directly phosphorylates Rim15 (Wanke et al., 2008). Tap42 phosphorylation is catalyzed by active TORC1 (Yan et al., 2006). Phosphorylated Tap42 interacts with TORC1 and associates with the catalytic subunit of type 2A phosphatases (PP2A) like Pph21 or Sit4 and thus inhibits their phosphatase activity (Di Como & Arndt, 1996; Jiang & Broach, 1999). Dissociation of the complex occurring in the case of TORC1 inactivity results in PP2A activation (Beck & Hall, 1999). PP2A dephosphorylates its downstream targets such as Gat1, Gln3. (Beck & Hall, 1999; Kuruvilla et al., 2001). This regulation is complex and different transcription factors are regulated differently depending on TORC1 stimuli (Broach, 2012; Conrad et al., 2014; Georis et al., 2009). In this model, we chose a reduced representation where Gat1 and Gln3 are dephosphorylated by active PP2A and either of them induces nitrogen catabolite repression (NCR) genes. Rtg1 and Rtg3 promote the expression of retrograde signaling (RTG) genes whose gene products enable alpha-ketoglutarate production and further processing into glutamine and glutamate to sustain amino acid biosynthesis (Liu & Butow, 1999). However, signaling via the RTG branch requires some additional regulation which involves Rtg1, 2 and 3 and the negative regulators Mks1 and Bmh1 and 2. In nitrogen-rich conditions, TORC1 phosphorylates Mks1 that complexes with the Bmh proteins thus sequestering Rtg1 and 3 in the cytoplasm. In contrast, nitrogen depletion causes reduced phosphorylation of Mks1 mediated by PP2A, so that the former complexes with Rtg2, consequently releasing Rtg1 and 3 into the nucleus where they act as transcriptional activators (Broach, 2012; Dilova et al., 2004). Again, we chose a reduced representation where TORC1 phosphorylates Mks1 and active PP2A dephosphorylates Mks1. Rtg1,3 is phosphorylated unless dephosphorylated Mks1 and Rtg2 are present. Then Rtg1,3 becomes dephosphorylated in the model and activates RTG transcription. TORC1-mediated phosphorylation of Sfp1 results in the transcription factor's nuclear translocation where it induces ribosomal protein and ribosome biogenesis gene expression (Lempiäinen et al., 2009; Marion et al., 2004). There is also a negative feedback mechanism in which phosphorylated Sfp1 negatively regulates the phosphorylation state of Sch9 (Lempiäinen et al., 2009). However, this negative feedback is not implemented in the model.

The described signaling activity may be valid for conditions in which enough glucose is available, and cells are not exposed to any stress factors. However, under glucose depletion, both branches downstream of TORC1, namely the PP2A and the Sch9 branch, show no or only little activity which is probably caused by crosstalk with the Snf1 pathway (Hughes Hallett et al., 2014). In this model, it is implemented in that way that phosphorylated Snf1 inhibits TORC1 activity as well as it phosphorylates Tap42.

## Crosstalk

To enable an efficient and fine-tuned adaption to environmental conditions such as different nutrient availabilities, interaction of the induced signaling pathways is required to integrate information. Although we integrated some of these crosstalk mechanisms we considered relevant for the model, many more pathway interactions were reported which highlights the fine-tuned regulation of integrating environmental changes. (Shashkova et al., 2015)

## Dynamics of the Boolean model indicate either a model gap or rate differences in the pathways.

When implementing the crosstalk, we found that the dynamics did not operate according to literature in contrast to the steady state result. In contrast to the literature, Adr1 was inactivated by PKA instead of Snf1(Cherry et al. 1989) and PKA acted as the main regulator of Rim15 instead of Sch9 (Ivo Pedruzzi et al. 2003). This could either indicate that these pathways may not operate on the same time scale or the complexity of the pathways is not equally known. When iterating over discrete time steps, one does not consider time but the complexity of the modeled pathways. For instance, if a pathway is well described and can be model in great detail, it takes many iterations to reach a steady state. In contrast, a poorly understood pathway may need very few iterations to reach the steady state although, in reality, signaling via the poorly understood pathway may take more steps than via the well-annotated pathway. In this work, a synchronous modeling scheme was used, meaning that at each iteration the state vectors are updated simultaneously as there is little information on the order and duration of state transitions available (Garg et al. 2008). In these cases, it is hard to say if the simulations reflect the reality, indicates that the PKA pathway is less understood than the Snf1 pathway or if the discrepancy is a result of a difference in rates between the pathways. Either way, these results highlight the lack of understanding of the dynamics of signal transduction in nutrient signaling pathways.

## Knock out of major signaling components reveals question marks about reported crosstalk mechanisms.

In the deletion experiments in the Boolean model during high nutrient availability, the simulation of Reg1 knockout showed almost the same effect on the SNF1 pathway as nutrient depletion. Only Adr1 activity was not affected which opposes the observations by Dombek et al. (1999) that described constitutive ADH2 expression in Reg1 mutant cells (Dombek et al. 1999). Since Adr1 is the main regulator of ADH2 activity, the previously discussed inhibition of Adr1 activity by active PKA (Cherry et al. 1989) may be the reason for these contradicting results and thus, the relevance of this crosstalk may be questioned.

In literature the Snf1 knockout is described to have a phenotype resembling over activation of PKA, however in our simulated deletion experiments in the Boolean model during low nutrient availability, AC was activated but the Krh proteins inhibit PKA if no glucose is present. PKA activation is a fine-tuned process that requires more complexity such as high cAMP concentrations upon Krh activation (T. Peeters et al., 2006) which could not be modeled using our Boolean approach.

# References

Bar-Peled, L., Chantranupong, L., Cherniack, A. D., Chen, W. W., Ottina, K. A., Grabiner, B. C., Spear, E. D., Carter, S. L., Meyerson, M., & Sabatini, D. M. (2013). A tumor suppressor complex with GAP activity for the Rag GTPases that signal amino acid sufficiency to mTORC1. *Science*, *340*(6136), 1100–1106. https://doi.org/10.1126/science.1232044

Barrett, L., Orlova, M., Maziarz, M., & Kuchin, S. (2012). Protein kinase a contributes to the negative control of SNF1 protein kinase in saccharomyces cerevisiae. *Eukaryotic Cell*, *11*(2), 119–128. https://doi.org/10.1128/EC.05061-11

Beck, T., & Hall, M. N. (1999). The TOR signalling pathway controls nuclear localization of nutrient- regulated transcription factors. *Nature*, *402*(6762), 689–692. https://doi.org/10.1038/45287

Binda, M., Péli-Gulli, M. P., Bonfils, G., Panchaud, N., Urban, J., Sturgill, T. W., Loewith, R., & De Virgilio, C. (2009). The Vam6 GEF Controls TORC1 by Activating the EGO Complex. *Molecular Cell*, *35*(5), 563–573. https://doi.org/10.1016/j.molcel.2009.06.033

Bonfils, G., Jaquenoud, M., Bontron, S., Ostrowicz, C., Ungermann, C., & De Virgilio, C. (2012). Leucyl-tRNA Synthetase Controls TORC1 via the EGO Complex. *Molecular Cell*, *46*(1), 105–110. https://doi.org/10.1016/j.molcel.2012.02.009

Broach, J. R. (2012). Nutritional control of growth and development in yeast. *Genetics*, *192*(1), 73–105. https://doi.org/10.1534/genetics.111.135731

Broek, D., Toda, T., Michaeli, T., Levin, L., Birchmeier, C., Zoller, M., Powers, S., & Wigler, M. (1987). The S. cerevisiae CDC25 gene product regulates the RAS/adenylate cyclase pathway. *Cell*, *48*(5), 789–799. https://doi.org/10.1016/0092-8674(87)90076-6

Castermans, D., Somers, I., Kriel, J., Louwet, W., Wera, S., Versele, M., Janssens, V., & Thevelein, J. M. (2012). Glucose-induced posttranslational activation of protein phosphatases PP2A and PP1 in yeast. *Cell Research*, *22*(6), 1058–1077. https://doi.org/10.1038/cr.2012.20

Cherry, J. R., Johnson, T. R., Dollard, C., Shuster, J. R., & Denis, C. L. (1989). Cyclic AMP-dependent protein kinase phosphorylates and inactivates the yeast transcriptional activator ADR1. *Cell*, *56*(3), 409–419. https://doi.org/10.1016/0092-8674(89)90244-4

Colombo, S., Ma, P., Cauwenberg, L., Winderickx, J., Crauwels, M., Teunissen, A., Nauwelaers, D., de Winde, J. H., Gorwa, M. F., Colavizza, D., & Thevelein, J. M. (1998). Involvement of distinct G-proteins, Gpa2 and Ras, in glucose- and intracellular acidification-induced cAMP signalling in the yeast Saccharomyces cerevisiae. *The EMBO Journal*, *17*(12), 3326–3341. https://doi.org/10.1093/emboj/17.12.3326

Conrad, M., Schothorst, J., Kankipati, H. N., Van Zeebroeck, G., Rubio-Texeira, M., & Thevelein, J. M. (2014). Nutrient sensing and signaling in the yeast Saccharomyces cerevisiae. In *FEMS Microbiology Reviews* (Vol. 38, Issue 2, pp. 254–299). Wiley-Blackwell. https://doi.org/10.1111/1574-6976.12065

Di Como, C. J., & Arndt, K. T. (1996). Nutrients, via the Tor proteins, stimulate the association of Tap42 with type 2A phosphatases. *Genes and Development*, *10*(15), 1904–1916. https://doi.org/10.1101/gad.10.15.1904

Dihazi, H., Kessler, R., & Eschrich, K. (2003). Glucose-induced stimulation of the Ras-cAMP pathway in yeast leads to multiple phosphorylations and activation of 6-phosphofructo-2-kinase. *Biochemistry*, *42*(20), 6275–6282. https://doi.org/10.1021/bi034167r

Dilova, I., Aronova, S., Chen, J. C. Y., & Powers, T. (2004). Tor signaling and nutrient-based signals converge on Mks1p phosphorylation to regulate expression of Rtg1p·Rtg3p-dependent target genes. *Journal of Biological Chemistry*, *279*(45), 46527–46535. https://doi.org/10.1074/jbc.M409012200

Dubouloz, F., Deloche, O., Wanke, V., Cameroni, E., & De Virgilio, C. (2005). The TOR and EGO protein complexes orchestrate microautophagy in yeast. *Molecular Cell*, *19*(1), 15–26. https://doi.org/10.1016/j.molcel.2005.05.020

Fernández-García, P., Peláez, R., Herrero, P., & Moreno, F. (2012). Phosphorylation of Yeast Hexokinase 2 Regulates Its Nucleocytoplasmic Shuttling *. *The Journal of Biological Chemistry*. https://doi.org/10.1074/jbc.M112.401679

Georis, I., Feller, A., Vierendeels, F., & Dubois, E. (2009). The Yeast GATA Factor Gat1 Occupies a Central Position in Nitrogen Catabolite Repression-Sensitive Gene Activation. *Molecular and Cellular Biology*, *29*(13), 3803–3815. https://doi.org/10.1128/mcb.00399-09

Hong, S. P., Leiper, F. C., Woods, A., Carling, D., & Carlson, M. (2003). Activation of yeast Snf1 and mammalian AMP-activated protein kinase by upstream kinases. *Proceedings of the National Academy of Sciences of the United States of America*, *100*(15), 8839–8843. https://doi.org/10.1073/pnas.1533136100

Hu, Y., Liu, E., Bai, X., & Zhang, A. (2010). The localization and concentration of the PDE2-encoded high-affinity cAMP phosphodiesterase is regulated by cAMP-dependent protein kinase A in the yeast Saccharomyces cerevisiae. *FEMS Yeast Research*, *10*(2), 177–187. https://doi.org/10.1111/j.1567-1364.2009.00598.x

Hughes Hallett, J. E., Luo, X., & Capaldi, A. P. (2014). State transitions in the TORC1 signaling pathway and information processing in Saccharomyces cerevisiae. *Genetics*, *198*(2), 773–786. https://doi.org/10.1534/genetics.114.168369

Jiang, Y., & Broach, J. R. (1999). Tor proteins and protein phosphatase 2A reciprocally regulate Tap42 in controlling cell growth in yeast. *The EMBO Journal*, *18*(10), 2782–2792. https://doi.org/10.1093/emboj/18.10.2782

Jones, S., Vignais, M. L., & Broach, J. R. (1991). The CDC25 protein of Saccharomyces cerevisiae promotes exchange of guanine nucleotides bound to ras. *Molecular and Cellular Biology*, *11*(5), 2641–2646. https://doi.org/10.1128/mcb.11.5.2641

Kacherovsky, N., Tachibana, C., Amos, E., Fox, D., & Young, E. T. (2008). Promoter binding by the Adr1 transcriptional activator may be regulated by phosphorylation in the DNA-binding region. *PLoS ONE*, *3*(9). https://doi.org/10.1371/journal.pone.0003213

Kataoka, T., Broek, D., & Wigler, M. (1985). DNA sequence and characterization of the S. cerevisiae gene encoding adenylate cyclase. *Cell*, *43*(2 PART 1), 493–505. https://doi.org/10.1016/0092-8674(85)90179-5

Kraakman, L., Lemaire, K., Ma, P., Teunlssen, A. W. R. H., Donaton, M. C. V., Van Dijck, P., Winderickx, J., De Winde, J. H., & Thevelein, J. M. (1999). A Saccharomyces cerevisiae G-protein coupled receptor, Gpr1, is specifically required for glucose activation of the cAMP pathway during the transition to growth on glucose. *Molecular Microbiology*, *32*(5), 1002–1012. https://doi.org/10.1046/j.1365-2958.1999.01413.x

Kuruvilla, F. G., Shamji, A. F., & Schreiber, S. L. (2001). Carbon- and nitrogen-quality signaling to translation are mediated by distinct GATA-type transcription factors. *Proceedings of the National Academy of Sciences of the United States of America*, *98*(13), 7283–7288. https://doi.org/10.1073/pnas.121186898

Lempiäinen, H., Uotila, A., Urban, J., Dohnal, I., Ammerer, G., Loewith, R., & Shore, D. (2009). Sfp1 Interaction with TORC1 and Mrs6 Reveals Feedback Regulation on TOR Signaling. *Molecular Cell*, *33*(6), 704–716. https://doi.org/10.1016/j.molcel.2009.01.034

Leverentz, M. K., & Reece, R. J. (2006). Phosphorylation of Zn(II)2Cys6 proteins: A cause or effect of transcriptional activation? *Biochemical Society Transactions*, *34*(5), 794–797. https://doi.org/10.1042/BST0340794

Liu, Z., & Butow, R. A. (1999). A Transcriptional Switch in the Expression of Yeast Tricarboxylic Acid Cycle Genes in Response to a Reduction or Loss of Respiratory Function. *Molecular and Cellular Biology*, *19*(10), 6720–6728. https://doi.org/10.1128/mcb.19.10.6720

Ludin, K., Jiang, R., & Carlson, M. (1998). Glucose-regulated interaction of a regulatory subunit of protein phosphatase 1 with the Snf1 protein kinase in Saccharomyces cerevisiae. *Proceedings of the National Academy of Sciences of the United States of America*, *95*(11), 6245–6250. https://doi.org/10.1073/pnas.95.11.6245

Ma, P., Wera, S., Van Dijck, P., & Thevelein, J. M. (1999). The PDE1-encoded low-affinity phosphodiesterase in the yeast Saccharomyces cerevisiae has a specific function in controlling agonist- induced cAMP signaling. *Molecular Biology of the Cell*, *10*(1), 91–104. https://doi.org/10.1091/mbc.10.1.91

MacPherson, S., Larochelle, M., & Turcotte, B. (2006). A Fungal Family of Transcriptional Regulators: the Zinc Cluster Proteins. *Microbiology and Molecular Biology Reviews*, *70*(3), 583–604. https://doi.org/10.1128/mmbr.00015-06

Marion, R. M., Regev, A., Segal, E., Barash, Y., Koller, D., Friedman, N., & O’Shea, E. K. (2004). Sfp1 is a stress- and nutrient-sensitive regulator of ribosomal protein gene expression. *Proceedings of the National Academy of Sciences of the United States of America*, *101*(40), 14315–14322. https://doi.org/10.1073/pnas.0405353101

Martínez-Pastor, M. T., Marchler, G., Schüller, C., Marchler-Bauer, A., Ruis, H., & Estruch, F. (1996). The Saccharomyces cerevisiae zinc finger proteins Msn2p and Msn4p are required for transcriptional induction through the stress response element (STRE). *The EMBO Journal*, *15*(9), 2227–2235. https://doi.org/10.1002/j.1460-2075.1996.tb00576.x

Matsumoto, K., Uno, I., Toh-E, A., Ishikawa, T., & Oshima, Y. (1982). Cyclic AMP may not be involved in catabolite repression in Saccharomyes cerevisiae: evidence from mutants capable of utilizing it as an adenine source. *Journal of Bacteriology*, *150*(1), 277–285. http://www.ncbi.nlm.nih.gov/pubmed/6277865

Nicastro, R., Tripodi, F., Gaggini, M., Castoldi, A., Reghellin, V., Nonnis, S., Tedeschi, G., & Coccetti, P. (2015). Snf1 phosphorylates adenylate cyclase and negatively regulates protein kinase A-dependent transcription in Saccharomyces cerevisiae. *Journal of Biological Chemistry*, *290*(41), 24715–24726. https://doi.org/10.1074/jbc.M115.658005

Nikawa, J., Sass, P., & Wigler, M. (1987). Cloning and characterization of the low-affinity cyclic AMP phosphodiesterase gene of Saccharomyces cerevisiae. *Molecular and Cellular Biology*, *7*(10), 3629–3636. https://doi.org/10.1128/mcb.7.10.3629

Pedruzzi, I., Bürckert, N., Egger, P., & De Virgilio, C. (2000). Saccharomyces cerevisiae Ras/cAMP pathway controls post-diauxic shift element-dependent transcription through the zinc finger protein Gis1. *The EMBO Journal*, *19*(11), 2569–2579. https://doi.org/10.1093/emboj/19.11.2569

Peeters, K., Van Leemputte, F., Fischer, B., Bonini, B. M., Quezada, H., Tsytlonok, M., Haesen, D., Vanthienen, W., Bernardes, N., Gonzalez-Blas, C. B., Janssens, V., Tompa, P., Versées, W., & Thevelein, J. M. (2017). Fructose-1,6-bisphosphate couples glycolytic flux to activation of Ras. *Nature Communications*, *8*(1). https://doi.org/10.1038/s41467-017-01019-z

Peeters, T., Louwet, W., Geladé, R., Nauwelaers, D., Thevelein, J. M., & Versele, M. (2006). Kelch-repeat proteins interacting with the Gα protein Gpa2 bypass adenylate cyclase for direct regulation of protein kinase A in yeast. *Proceedings of the National Academy of Sciences of the United States of America*, *103*(35), 13034–13039. https://doi.org/10.1073/pnas.0509644103

Portela, P., Howell, S., Moreno, S., & Rossi, S. (2002). In vivo and in vitro phosphorylation of two isoforms of yeast pyruvate kinase by protein kinase A. *Journal of Biological Chemistry*, *277*(34), 30477–30487. https://doi.org/10.1074/jbc.M201094200

Reinke, A., Anderson, S., McCaffery, J. M., Yates, J., Aronova, S., Chu, S., Fairclough, S., Iverson, C., Wedaman, K. P., & Powers, T. (2004). TOR Complex 1 Includes a Novel Component, Tco89p (YPL180w), and Cooperates with Ssd1p to Maintain Cellular Integrity in Saccharomyces cerevisiae. *Journal of Biological Chemistry*, *279*(15), 14752–14762. https://doi.org/10.1074/jbc.M313062200

Rittenhouse, J., Moberly, L., & Marcus, F. (1987). Phosphorylation in vivo of yeast (Saccharomyces cerevisiae) fructose-1,6-bisphosphatase at the cyclic AMP-dependent site. *Journal of Biological Chemistry*, *262*(21), 10114–10119.

Robinson, L. C., Gibbs, J. B., Marshall, M. S., Sigal, I. S., & Tatchell, K. (1987). CDC25: A component of the RAS-adenylate cyclase pathway in Saccharomyces cerevisiae. *Science*, *235*(4793), 1218–1221. https://doi.org/10.1126/science.3547648

Rolland, F., De Winde, J. H., Lemaire, K., Boles, E., Thevelein, J. M., & Winderickx, J. (2000). Glucose-induced cAMP signalling in yeast requires both a G-protein coupled receptor system for extracellular glucose detection and a separable hexose kinase-dependent sensing process. *Molecular Microbiology*, *38*(2), 348–358. https://doi.org/10.1046/j.1365-2958.2000.02125.x

Santangelo, G. M. (2006). Glucose Signaling in Saccharomyces cerevisiae. *Microbiology and Molecular Biology Reviews*, *70*(1), 253–282. https://doi.org/10.1128/mmbr.70.1.253-282.2006

Sanz, P., Alms, G. R., Haystead, T. A. J., & Carlson, M. (2000). Regulatory Interactions between the Reg1-Glc7 Protein Phosphatase and the Snf1 Protein Kinase. *Molecular and Cellular Biology*, *20*(4), 1321–1328. https://doi.org/10.1128/mcb.20.4.1321-1328.2000

Sass, P., Field, J., Nikawa, J., Toda, T., & Wigler, M. (1986). Cloning and characterization of the high-affinity cAMP phosphodiesterase of Saccharomyces cerevisiae. *Proceedings of the National Academy of Sciences of the United States of America*, *83*(24), 9303–9307. https://doi.org/10.1073/pnas.83.24.9303

Schepers, W., Van Zeebroeck, G., Pinkse, M., Verhaert, P., & Thevelein, J. M. (2012). In vivo phosphorylation of Ser21 and Ser83 during nutrient-induced activation of the yeast protein kinase A (PKA) target trehalase. *Journal of Biological Chemistry*, *287*(53), 44130–44142. https://doi.org/10.1074/jbc.M112.421503

Schüller, H. J. (2003). Transcriptional control of nonfermentative metabolism in the yeast Saccharomyces cerevisiae. *Current Genetics*, *43*(3), 139–160. https://doi.org/10.1007/s00294-003-0381-8

Shashkova, S., Welkenhuysen, N., & Hohmann, S. (2015). Molecular communication: crosstalk between the Snf1 and other signaling pathways. *FEMS Yeast Research*, *15*. https://doi.org/10.1093/femsyr/fov026

Smith, J. J., Miller, L. R., Kreisberg, R., Vazquez, L., Wan, Y., & Aitchison, J. D. (2011). Environment-responsive transcription factors bind subtelomeric elements and regulate gene silencing. *Molecular Systems Biology*, *7*, 455. https://doi.org/10.1038/msb.2010.110

Soontorngun, N., Baramee, S., Tangsombatvichit, C., Thepnok, P., Cheevadhanarak, S., Robert, F., & Turcotte, B. (2012). Genome-wide location analysis reveals an important overlap between the targets of the yeast transcriptional regulators Rds2 and Adr1. *Biochemical and Biophysical Research Communications*, *423*(4), 632–637. https://doi.org/10.1016/j.bbrc.2012.05.151

Sutherland, C. M., Hawley, S. A., McCartney, R. R., Leech, A., Stark, M. J. R., Schmidt, M. C., & Hardie, D. G. (2003). Elm1p is one of three upstream kinases for the Saccharomyces cerevisiae SNF1 complex. *Current Biology : CB*, *13*(15), 1299–1305. https://doi.org/10.1016/s0960-9822(03)00459-7

Swinnen, E., Wanke, V., Roosen, J., Smets, B., Dubouloz, F., Pedruzzi, I., Cameroni, E., De Virgilio, C., & Winderickx, J. (2006). Rim15 and the crossroads of nutrient signalling pathways in Saccharomyces cerevisiae. In *Cell Division* (Vol. 1, p. 3). BioMed Central. https://doi.org/10.1186/1747-1028-1-3

Tanaka, K, Matsumoto, K., & Toh-E, A. (1989). IRA1, an inhibitory regulator of the RAS-cyclic AMP pathway in Saccharomyces cerevisiae. *Molecular and Cellular Biology*, *9*(2), 757–768. https://doi.org/10.1128/mcb.9.2.757

Tanaka, K, Nakafuku, M., Tamanoi, F., Kaziro, Y., Matsumoto, K., & Toh-e, A. (1990). IRA2, a second gene of Saccharomyces cerevisiae that encodes a protein with a domain homologous to mammalian ras GTPase-activating protein. *Molecular and Cellular Biology*, *10*(8), 4303–4313. https://doi.org/10.1128/mcb.10.8.4303

Tanaka, Kazuma, Nakafuku, M., Satoh, T., Marshall, M. S., Gibbs, J. B., Matsumoto, K., Kaziro, Y., & Toh-e, A. (1990). S. cerevisiae genes IRA1 and IRA2 encode proteins that may be functionally equivalent to mammalian ras GTPase activating protein. *Cell*, *60*(5), 803–807. https://doi.org/10.1016/0092-8674(90)90094-U

Toda, T, Cameron, S., Sass, P., Zoller, M., Scott, J. D., McMullen, B., Hurwitz, M., Krebs, E. G., & Wigler, M. (1987). Cloning and characterization of BCY1, a locus encoding a regulatory subunit of the cyclic AMP-dependent protein kinase in Saccharomyces cerevisiae. *Molecular and Cellular Biology*, *7*(4), 1371–1377. https://doi.org/10.1128/mcb.7.4.1371

Toda, Takashi, Cameron, S., Sass, P., Zoller, M., & Wigler, M. (1987). Three different genes in S. cerevisiae encode the catalytic subunits of the cAMP-dependent protein kinase. *Cell*, *50*(2), 277–287. https://doi.org/10.1016/0092-8674(87)90223-6

Toda, Takashi, Uno, I., Ishikawa, T., Powers, S., Kataoka, T., Broek, D., Cameron, S., Broach, J., Matsumoto, K., & Wigler, M. (1985). In yeast, RAS proteins are controlling elements of adenylate cyclase. *Cell*, *40*(1), 27–36. https://doi.org/10.1016/0092-8674(85)90305-8

Turcotte, B., Liang, X. B., Robert, F., & Soontorngun, N. (2010). Transcriptional regulation of nonfermentable carbon utilization in budding yeast. In *FEMS Yeast Research* (Vol. 10, Issue 1, pp. 2–13). PMC Canada manuscript submission. https://doi.org/10.1111/j.1567-1364.2009.00555.x

Urban, J., Soulard, A., Huber, A., Lippman, S., Mukhopadhyay, D., Deloche, O., Wanke, V., Anrather, D., Ammerer, G., Riezman, H., Broach, J. R., De Virgilio, C., Hall, M. N., & Loewith, R. (2007). Sch9 Is a Major Target of TORC1 in Saccharomyces cerevisiae. *Molecular Cell*, *26*(5), 663–674. https://doi.org/10.1016/j.molcel.2007.04.020

Wanke, V., Cameroni, E., Uotila, A., Piccolis, M., Urban, J., Loewith, R., & De Virgilio, C. (2008). Caffeine extends yeast lifespan by targeting TORC1. *Molecular Microbiology*, *69*(1), 277–285. https://doi.org/10.1111/j.1365-2958.2008.06292.x

Westholm, J. O., Nordberg, N., Murén, E., Ameur, A., Komorowski, J., & Ronne, H. (2008). Combinatorial control of gene expression by the three yeast repressors Mig1, Mig2 and Mig3. *BMC Genomics*, *9*(SUPPL. 2), 601. https://doi.org/10.1186/1471-2164-9-601

Woods, A., Munday, M. R., Scott, J., Yang, X., Carlson, M., & Carling, D. (1994). Yeast SNF1 is functionally related to mammalian AMP-activated protein kinase and regulates acetyl-CoA carboxylase in vivo. *Journal of Biological Chemistry*, *269*(30), 19509–19515.

Yan, G., Shen, X., & Jiang, Y. (2006). Rapamycin activates Tap42-associated phosphatases by abrogating their association with Tor complex 1. *EMBO Journal*, *25*(15), 3546–3555. https://doi.org/10.1038/sj.emboj.7601239
