# Supplementary material for: A novel yeast hybrid modeling framework integrating Boolean and enzyme-constrained networks enables exploration of the interplay between signaling and metabolism
Source: PLoS Comput Biol. 2021 Apr 9;17(4):e1008891. doi: 10.1371/journal.pcbi.1008891 (PMC8059808; doi:10.1371/journal.pcbi.1008891)
Supplement: S2 Text — Includes detailed information on the analysis of protein prediction and deletion strain simulations. (DOCX) [file pcbi.1008891.s002.docx]

Supporting Information on the hybrid model

A novel yeast hybrid modeling framework integrating Boolean and enzyme-constrained networks enables exploration of the interplay between signaling and metabolism

Linnea Österberg^1,2,3^, Iván Domenzain ^3,4^, Julia Münch^1,2^, Jens Nielsen^3,4,5^, Stefan Hohmann ^3^, Marija Cvijovic^1,2*^

^1^ Department of Mathematical Sciences, University of Gothenburg, Gothenburg, Sweden

^2^ Department of Mathematical Sciences, Chalmers University of Technology, Gothenburg, Sweden

^3^ Department of Biology and Biological Engineering, Chalmers University of Technology, Gothenburg, Sweden

^4^ Novo Nordisk Foundation Center for Biosustainability, Chalmers University of Technology, SE41296 Gothenburg, Sweden

^5^ BioInnovation Institute, Ole Maaløes Vej 3, DK2200 Copenhagen, Denmark

*** Correspondence:**

Marija Cvijovic
[marija.cvijovic@chalmers.se](mailto:marija.cvijovic@chalmers.se)

# Hybrid model

The ecModel predicts 48.3% of the proteins within one order of magnitude in the respiration state and 32.5% of the proteins in the fermentation state compared to the hybrid models 65.5% and 40.8% respectively. Proteins, miss-predicted with over 5 orders of magnitude, are either not detected in the experimental data but used by the models or proteins not used by the models but detected by experimental data. This is mainly proteins of the pathways that make out the precursors for the biomass, proteins of the oxidative phosphorylation pathway and isoenzymes. This can largely be explained by the models having a preferential use of isoenzymes, pathways not necessary for growth in a specific steady state are not used and that membrane proteins are often not detected in mass spectrometry studies. By adding the signaling layer to the ecModel we force the use of isoenzymes and pathways that are not used in that particular steady state which decreases the number of miss predicted proteins with over 5 orders of magnitude from 32.2% to 13.7% in the respiration state and from 35.0% to 17.5% in the fermentation state. The other miss predicted proteins, were on average miss predicted by 1.80 orders of magnitude by the ecModel in the respiration state and by 1.71 orders of magnitude by the hybrid model. In the fermentation state, the ecModel was off by on average 1.49 orders of magnitude whereas the hybrid model was off by 1.61.

One of the contributing factors to the miss-predicted proteins could be the kcat curating process. The kcats are curated through GECKO; in case the kcats are not available for *Saccharomyces cerevisiae* the kcats are taken from the phylogenetically closest related organism that the value does exist for. This process will make sure we have some constraints based on enzyme kinetics but will also contribute to errors in our predictions where the values differ between organisms. Also, predicting proteins based on optimality principles has the inherent problem that when confronted with several choices of how to carry a flux trough a reaction, the determining factor will lie heavily on the molecular weight of the proteins involved in the reaction and the flux needed based on the steady state assumption. In reality, cells need to be prepared for changes in the environment and are seldom in a steady state, meaning they also need to put energy into enzymes not needed for the specific conditions. We also see a trend in both respiration and fermentation that the glycolysis, TCA and PPP proteins are overpredicted while the OPP proteins are underpredicted. This might be due to two factors, glycolysis, TCA and PPP mainly consist of globular proteins and the OPP proteins are mainly membrane-bound. There is an intrinsic difficulty in mass spectrometry studies to quantify membrane proteins which might contribute to the appearance of a higher relative abundance of globular proteins compared to membrane-bound proteins.
