# Supplementary figures and images for: A novel yeast hybrid modeling framework integrating Boolean and enzyme-constrained networks enables exploration of the interplay between signaling and metabolism
Source: PLoS Comput Biol. 2021 Apr 9;17(4):e1008891. doi: 10.1371/journal.pcbi.1008891 (PMC8059808; doi:10.1371/journal.pcbi.1008891)

**A** KO Glucose{0} Nitrogen{0}

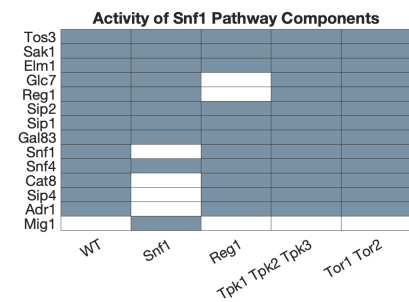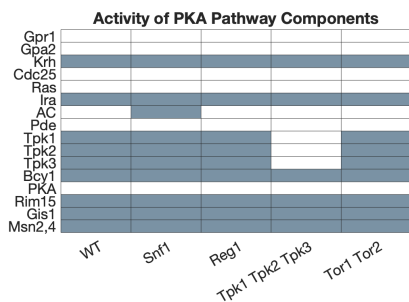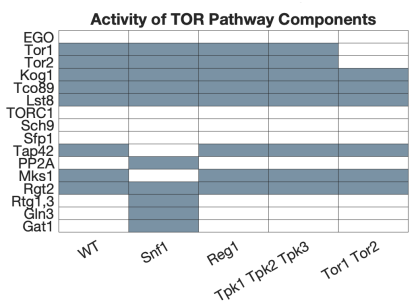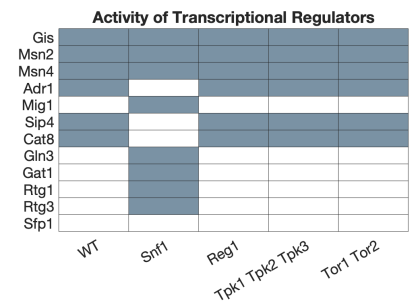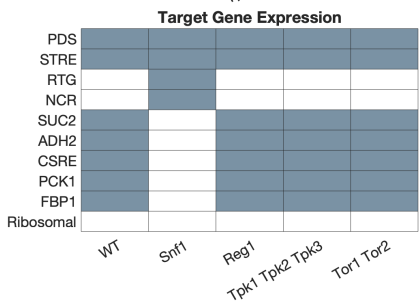

**B** KO Glucose{1} Nitrogen{1}

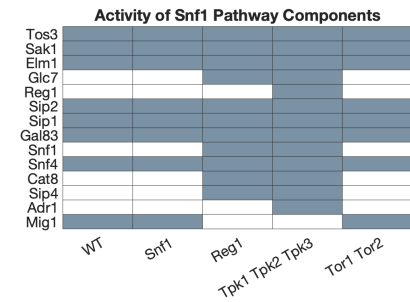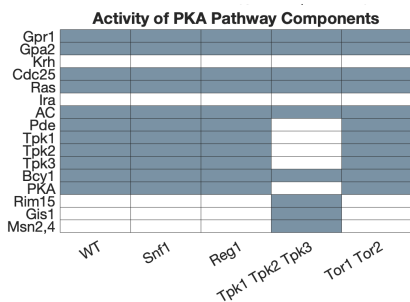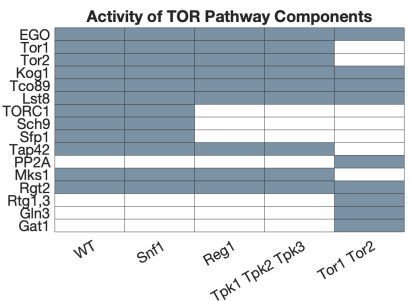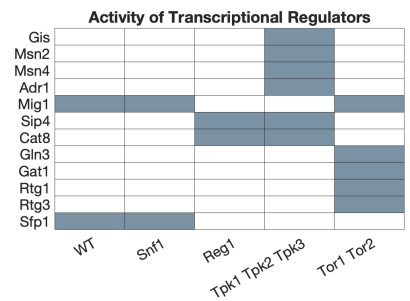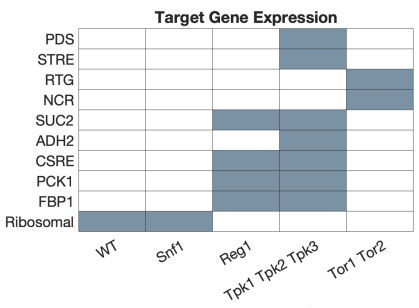

Supplement: S2 Fig — Panel (A) shows the KO behavior in low nutrient conditions compared to the WT and panel (B) show the KO behavior in high nutrient conditions compared to the WT (PDF) [file pcbi.1008891.s004.pdf]

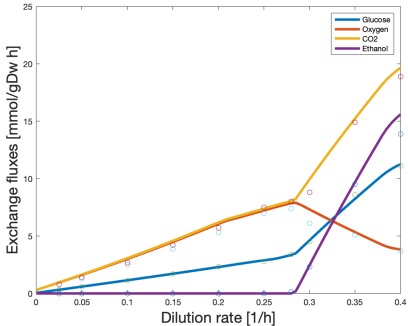

Supplement: S3 Fig — Simulations showed a median relative error of 9.82% in the whole range of dilution rates from 0 to 0.4 h-1. (PDF) [file pcbi.1008891.s005.pdf]
