# Supplementary material for: A novel yeast hybrid modeling framework integrating Boolean and enzyme-constrained networks enables exploration of the interplay between signaling and metabolism
Source: PLoS Comput Biol. 2021 Apr 9;17(4):e1008891. doi: 10.1371/journal.pcbi.1008891 (PMC8059808; doi:10.1371/journal.pcbi.1008891)
Supplement: S1 Table — (DOCX) [file pcbi.1008891.s007.docx]

S1 Table. Rules and references associated to any field of any of the Boolean vectors in the Boolean module.

| SNF1 | | | | | |
| --- | --- | --- | --- | --- | --- |
| Name | Presence | | | Phosphorylation | Specific activity |
| Tos3 | Present unless deleted | | | - | - |
| Sak1 | Present unless deleted | | | - | - |
| Elm1 | Present unless deleted | | | - | - |
| Glc7 | Present unless deleted | | | - | Active when Glc7 is present and Reg1 is present and phosphorylated.  Inactive when Reg1 is unphosphorylated and Glc7 is present or when Glc7 is not present. (Sanz et al., 2000) |
| Reg1 | Present unless deleted | | | Phosphorylated when Glc7, Reg1, Snf4 and any of Sip2, Sip1 or Gal83 is present as well as Snf1 is phosphorylated and Reg1 is not phosphorylated or if Hxk2 is phosphorylated(Sanz et al., 2000).  Unphosphorylated when Hxk2 and Snf1 is not phosphorylated and Glc7 is present, active and Reg1 is present and phosphorylated(Sanz et al., 2000). | - |
| Sip2 | Present unless deleted | | | - | - |
| Sip1 | Present unless deleted | | | - | - |
| Gal83 | Present unless deleted | | | - | - |
| Snf1 | Present unless deleted | | | Phosphorylated when glucose is absent, Snf1 is present, any of Tos3, Elm1 or Sak1 is present and, Snf1 and Reg1 is unphosphorylated (Hong et al., 2003; Sanz et al., 2000; Sutherland et al., 2003). Unphosphorylated when Snf1 is not present.  Unphosphorylated when Glucose, Glc7 and Reg1 is present, PKA and Glc7 is active and Reg1 and Snf1 is phosphorylated (Sanz et al., 2000) (Barrett et al., 2012; Castermans et al., 2012) | - |
| Snf4 | Present unless deleted | | | - | - |
| Cat8 | Present unless deleted | | | Always unphosphorylated unless Snf1 is active and Snf4 and Cat8 is present as well as any of Sip2, Sip1 or Gal83. (Broach, 2012; Leverentz & Reece, 2006; MacPherson et al., 2006; Turcotte et al., 2010). | - |
| Sip4 | Present unless deleted | | | Always unphosphorylated unless Snf1 is active and Snf4 and Sip4 is present as well as any of Sip2, Sip1 or Gal83. (Broach, 2012; Leverentz & Reece, 2006; MacPherson et al., 2006; Turcotte et al., 2010). | - |
| Adr1 | Present unless deleted | | | Always unphosphorylated unless Snf1 is active and Snf4 and Adr1 is present as well as any of Sip2, Sip1 or Gal83. (Broach, 2012; Kacherovsky et al., 2008; Smith et al., 2011; Soontorngun et al., 2012; Turcotte et al., 2010) | Active if precent, phosphorylated and PKA is active (Cherry et al., 1989). |
| Mig1 | Present unless deleted | | | Always unphosphorylated unless Snf1 is active and Snf4 and Mig1 is present as well as any of Sip2, Sip1 or Gal83. (Broach, 2012; Santangelo, 2006; Schüller, 2003; Westholm et al., 2008) | - |
| PKA pathway | | | | | |
| Name | Presence | | | Phosphorylation | Specific activity |
| Gpr1 | Present unless deleted | | | - | Always inactive unless GLUex and Gpr1 are present. (Colombo et al., 1998; Kraakman et al., 1999). |
| Gpa2 | Present unless deleted | | | - | Active if Gpr1 is active and Gpa2 is present.  Inactive if Gpr1 is inactive or Gpa2 is deleted.(Colombo et al., 1998; Kraakman et al., 1999). |
| Krh | Present unless deleted | | | - | Always active unless deleted or Gpa2 is active. (T. Peeters et al., 2006) |
| Cdc25 | Present unless deleted | | | - | Always inactive unless the metabolite F16BP and pathway component Cdc25 are present. (K. Peeters et al., 2017). |
| Ras | Present unless deleted | | | - | Active when Ras is present, Cdc25 is active and Ira is inactive (and Ras was previous inactive).  Inactive when Ras is present, Cdc25 is inactive and Ira is active (and Ras is previously active). (Broek et al., 1987; Jones et al., 1991; Robinson et al., 1987; K Tanaka et al., 1989, 1990; Kazuma Tanaka et al., 1990). |
| Ira | Present unless deleted | | | - | Always inactive unless GLUex and Ira are present. |
| AC | Present unless deleted | | | - | Active when Ras is active and AC is present, or when Ras and Gpa2 is active and AC is present.(Kataoka et al., 1985; Rolland et al., 2000; Takashi Toda et al., 1985)  Inactive if AC is deleted.  Inactive when Gpa2 and Ras is inactive, AC, Snf4 is present and Sip2, Sip1 or Gal83 is present and Snf1 is phosphorylated. (Nicastro et al., 2015) |
| Pde | Present unless deleted | | | Always unphosphorylated unless Pde is present and PKA is active. (Hu et al., 2010; Ma et al., 1999; Nikawa et al., 1987; Sass et al., 1986). | - |
| Tpk1 | Present unless deleted | | | - | - |
| Tpk2 | Present unless deleted | | | - | - |
| Tpk3 | Present unless deleted | | | - | - |
| Bcy1 | Present unless deleted | | | - | - |
| PKA | Always absent unless Tpk1, Tpk2 or Tpk3 is present. (Matsumoto et al., 1982; T Toda et al., 1987; Takashi Toda et al., 1987). | | | - | Active if PKA, Bcy1 and cAMP is present and Krh is inactive or Bcy1 is deleted.  Inactive if PKA and Bcy1 is present at the same time as Krh is active or cAMP or PKA is absent.(T. Peeters et al., 2006; T Toda et al., 1987; Takashi Toda et al., 1987) |
| Rim15 | Present unless deleted | | | Always unphosphorylated unless PKA is active and Rim15 is present. (Swinnen et al., 2006).  Phosphorylated if Rim15 is present and Sch9 is phosphorylated. (Wanke et al., 2008). | - |
| Gis1 | Present unless deleted | | | Always unphosphorylated unless Rim15 is unphosphorylated and Gis1 is present.(Swinnen et al., 2006). | - |
| Msn2,4 | Present unless deleted | | | Always unphosphorylated unless Rim15 is unphosphorylated and Msn2,4 is present.(Swinnen et al., 2006). | - |
| TOR | | | | | |
| Name | Presence | | | Phosphorylation | Specific activity |
| EGO | Present unless deleted | | | - | Always inactive unless NH3 and EGO is present. (Bar-Peled et al., 2013; Binda et al., 2009; Bonfils et al., 2012) |
| Tor1 | Present unless deleted | | | - | - |
| Tor2 | Present unless deleted | | | - | - |
| Kog1 | Present unless deleted | | | - | - |
| Tco89 | Present unless deleted | | | - | - |
| Lst8 | Present unless deleted | | | - | - |
| TORC1 | Always absent unless if Tor1 or Tor2 is present as well as Kog1, Toc89 and Lst8. (Reinke et al., 2004) | | | - | Always inactive unless EGO is active and TORC1 is present. (Binda et al., 2009)  Inactive if Snf1 is phosphorylated and EGO is active and TORC1 is present. (Hughes Hallett et al., 2014). |
| Sch9 | Present unless deleted | | | Always unphosphorylated unless TORC1 is active and Sch9 is present. (Urban et al., 2007) | - |
| Sfp1 | Present unless deleted | | | Always unphosphorylated unless TORC1 is active and Sfp1 is present. (Lempiäinen et al., 2009; Marion et al., 2004). | - |
| Tap42 | Present unless deleted | | | Phosphorylated when TORC1 is active and Tap42 is present.  Unphosphorylated if Tap42 is present and TORC1 is inactive or if Tap42 is absent. (Beck & Hall, 1999). (Di Como & Arndt, 1996; Jiang & Broach, 1999). (Yan et al., 2006).  Phosphorylated when Snf1 is phosphorylated and Tap42 is present. (Hughes Hallett et al., 2014).  Unphosphorylated if Tap42 is present, TORC1 is inactive and Snf1 is unphosphorylated. (Hughes Hallett et al., 2014). | - |
| PP2A | Present unless deleted | | | - | Always active unless Tap42 is phosphorylated and present and PPA2 is present or if PPA2 is absent. (Beck & Hall, 1999). (Di Como & Arndt, 1996; Jiang & Broach, 1999). (Yan et al., 2006). |
| Mks1 | Present unless deleted | | | Phosphorylated if TORC1 is active and Mks1 is present, or if GLUex is absent and Mks1 is present. (Hughes Hallett et al., 2014).  Unphosphorylated if Mks1 and PP2A is present and PP2A is active or if Mks1 is absent. (Broach, 2012; Dilova et al., 2004). | - |
| Rtg2 | Present unless deleted | | | - | - |
| Rtg1,3 | Present unless deleted | | | Always phosphorylated unless Mks1, Rtg2 and Rtg1,3 is present and Mks1 is unphosphorylated. (Broach, 2012; Dilova et al., 2004). | - |
| Gln3 | Present unless deleted | | | Always phosphorylated unless PP2A is present and phosphorylated and Gln3 is present. (Broach, 2012; Conrad et al., 2014; Georis et al., 2009). | - |
| Gat1 | Present unless deleted | | | Always phosphorylated unless PP2A is present and phosphorylated and Gat1 is present. (Broach, 2012; Conrad et al., 2014; Georis et al., 2009). | - |
| Enzymes | | | | | |
| Name | Presence | | | Phosphorylation | |
| PFK2 | Present unless deleted | | | Always unphosphorylated unless PKA is active and PFK2 is present. (Dihazi et al., 2003) (Active when phosphorylated.) | |
| TREH | Present unless deleted | | | Always unphosphorylated unless PKA is active and TREH is present.(Schepers et al., 2012) | |
| PK | Present unless deleted | | | Always unphosphorylated unless PKA is active and PK is present. (Portela et al., 2002) | |
| FBP1 | Present unless deleted. Present is FBP1 is active as target. | | | Always unphosphorylated unless PKA is active and FBP1 is present. (Rittenhouse et al., 1987). | |
| ACC | Present unless deleted | | | Always unphosphorylated unless Snf1 is active and Snf4 and ACC is present as well as any of Sip2, Sip1 or Gal83.(Woods et al., 1994) | |
| HXK2 | Present unless deleted | | | Always unphosphorylated unless HXK2 is present and glucose is not present. (Fernández-García et al., 2012) | |
| Metabolites | | | | | |
| Name | | | Presence | | |
| GLUex | | | 1 | | |
| ATP | | | Always present unless deleted. | | |
| cAMP | | | Present when ATP is present and AC is active.  Absent when AC is inactive and PDE is present and phosphorylated. (Hu et al., 2010; Ma et al., 1999; Nikawa et al., 1987; Sass et al., 1986) | | |
| F16BP | | | Always absent unless GLUex is present. | | |
| NH3 | | | 1 | | |
| Targets | | | | | |
| Name | | Activity | | | |
| PDS | | Always inactive unless Gis1 is phosphorylated. (Martínez-Pastor et al., 1996; Pedruzzi et al., 2000). | | | |
| STRE | | Always inactive unless Msn2,4 is phosphorylated. (Martínez-Pastor et al., 1996; Pedruzzi et al., 2000). | | | |
| RTG | | Always inactive unless Rtg1,3 is unphosphorylated and present. (Broach, 2012; Dilova et al., 2004; Liu & Butow, 1999) | | | |
| NCR | | Always inactive unless Gat1 is present but not phosphorylated or is Gln3 is present but not phosphorylated. (Broach, 2012; Conrad et al., 2014; Georis et al., 2009). | | | |
| SUC2 | | Always inactive unless Mig1 is phosphorylated(Broach, 2012; Santangelo, 2006; Schüller, 2003; Westholm et al., 2008). | | | |
| ADH2 | | Inactive if Adr1 is phosphorylated and PKA is active. Always inactive unless Adr1 is phosphorylated. (Broach, 2012; Cherry et al., 1989; Kacherovsky et al., 2008; Smith et al., 2011; Soontorngun et al., 2012; Turcotte et al., 2010) | | | |
| CSRE | | Always inactive unless Sip1 or Cat8 is phosphorylated. (Broach, 2012; Leverentz & Reece, 2006; MacPherson et al., 2006; Turcotte et al., 2010). | | | |
| PCK1 | | Always inactive unless Sip1 or Cat8 is phosphorylated. (Broach, 2012; Leverentz & Reece, 2006; MacPherson et al., 2006; Turcotte et al., 2010). | | | |
| FBP1 | | Always inactive unless Sip1 or Cat8 is phosphorylated. (Broach, 2012; Leverentz & Reece, 2006; MacPherson et al., 2006; Turcotte et al., 2010). | | | |
| Ribosomal | | Always inactive unless Sfp1 is phosphorylated. (Lempiäinen et al., 2009; Marion et al., 2004). | | | |

**References**

Bar-Peled, L., Chantranupong, L., Cherniack, A. D., Chen, W. W., Ottina, K. A., Grabiner, B. C., Spear, E. D., Carter, S. L., Meyerson, M., & Sabatini, D. M. (2013). A tumor suppressor complex with GAP activity for the Rag GTPases that signal amino acid sufficiency to mTORC1. *Science*, *340*(6136), 1100–1106. https://doi.org/10.1126/science.1232044

Barrett, L., Orlova, M., Maziarz, M., & Kuchin, S. (2012). Protein kinase a contributes to the negative control of SNF1 protein kinase in saccharomyces cerevisiae. *Eukaryotic Cell*, *11*(2), 119–128. https://doi.org/10.1128/EC.05061-11

Beck, T., & Hall, M. N. (1999). The TOR signalling pathway controls nuclear localization of nutrient- regulated transcription factors. *Nature*, *402*(6762), 689–692. https://doi.org/10.1038/45287

Binda, M., Péli-Gulli, M. P., Bonfils, G., Panchaud, N., Urban, J., Sturgill, T. W., Loewith, R., & De Virgilio, C. (2009). The Vam6 GEF Controls TORC1 by Activating the EGO Complex. *Molecular Cell*, *35*(5), 563–573. https://doi.org/10.1016/j.molcel.2009.06.033

Bonfils, G., Jaquenoud, M., Bontron, S., Ostrowicz, C., Ungermann, C., & De Virgilio, C. (2012). Leucyl-tRNA Synthetase Controls TORC1 via the EGO Complex. *Molecular Cell*, *46*(1), 105–110. https://doi.org/10.1016/j.molcel.2012.02.009

Broach, J. R. (2012). Nutritional control of growth and development in yeast. *Genetics*, *192*(1), 73–105. https://doi.org/10.1534/genetics.111.135731

Broek, D., Toda, T., Michaeli, T., Levin, L., Birchmeier, C., Zoller, M., Powers, S., & Wigler, M. (1987). The S. cerevisiae CDC25 gene product regulates the RAS/adenylate cyclase pathway. *Cell*, *48*(5), 789–799. https://doi.org/10.1016/0092-8674(87)90076-6

Castermans, D., Somers, I., Kriel, J., Louwet, W., Wera, S., Versele, M., Janssens, V., & Thevelein, J. M. (2012). Glucose-induced posttranslational activation of protein phosphatases PP2A and PP1 in yeast. *Cell Research*, *22*(6), 1058–1077. https://doi.org/10.1038/cr.2012.20

Cherry, J. R., Johnson, T. R., Dollard, C., Shuster, J. R., & Denis, C. L. (1989). Cyclic AMP-dependent protein kinase phosphorylates and inactivates the yeast transcriptional activator ADR1. *Cell*, *56*(3), 409–419. https://doi.org/10.1016/0092-8674(89)90244-4

Colombo, S., Ma, P., Cauwenberg, L., Winderickx, J., Crauwels, M., Teunissen, A., Nauwelaers, D., de Winde, J. H., Gorwa, M. F., Colavizza, D., & Thevelein, J. M. (1998). Involvement of distinct G-proteins, Gpa2 and Ras, in glucose- and intracellular acidification-induced cAMP signalling in the yeast Saccharomyces cerevisiae. *The EMBO Journal*, *17*(12), 3326–3341. https://doi.org/10.1093/emboj/17.12.3326

Conrad, M., Schothorst, J., Kankipati, H. N., Van Zeebroeck, G., Rubio-Texeira, M., & Thevelein, J. M. (2014). Nutrient sensing and signaling in the yeast Saccharomyces cerevisiae. In *FEMS Microbiology Reviews* (Vol. 38, Issue 2, pp. 254–299). Wiley-Blackwell. https://doi.org/10.1111/1574-6976.12065

Di Como, C. J., & Arndt, K. T. (1996). Nutrients, via the Tor proteins, stimulate the association of Tap42 with type 2A phosphatases. *Genes and Development*, *10*(15), 1904–1916. https://doi.org/10.1101/gad.10.15.1904

Dihazi, H., Kessler, R., & Eschrich, K. (2003). Glucose-induced stimulation of the Ras-cAMP pathway in yeast leads to multiple phosphorylations and activation of 6-phosphofructo-2-kinase. *Biochemistry*, *42*(20), 6275–6282. https://doi.org/10.1021/bi034167r

Dilova, I., Aronova, S., Chen, J. C. Y., & Powers, T. (2004). Tor signaling and nutrient-based signals converge on Mks1p phosphorylation to regulate expression of Rtg1p·Rtg3p-dependent target genes. *Journal of Biological Chemistry*, *279*(45), 46527–46535. https://doi.org/10.1074/jbc.M409012200

Fernández-García, P., Peláez, R., Herrero, P., & Moreno, F. (2012). Phosphorylation of Yeast Hexokinase 2 Regulates Its Nucleocytoplasmic Shuttling *. *The Journal of Biological Chemistry*. https://doi.org/10.1074/jbc.M112.401679

Georis, I., Feller, A., Vierendeels, F., & Dubois, E. (2009). The Yeast GATA Factor Gat1 Occupies a Central Position in Nitrogen Catabolite Repression-Sensitive Gene Activation. *Molecular and Cellular Biology*, *29*(13), 3803–3815. https://doi.org/10.1128/mcb.00399-09

Hong, S. P., Leiper, F. C., Woods, A., Carling, D., & Carlson, M. (2003). Activation of yeast Snf1 and mammalian AMP-activated protein kinase by upstream kinases. *Proceedings of the National Academy of Sciences of the United States of America*, *100*(15), 8839–8843. https://doi.org/10.1073/pnas.1533136100

Hu, Y., Liu, E., Bai, X., & Zhang, A. (2010). The localization and concentration of the PDE2-encoded high-affinity cAMP phosphodiesterase is regulated by cAMP-dependent protein kinase A in the yeast Saccharomyces cerevisiae. *FEMS Yeast Research*, *10*(2), 177–187. https://doi.org/10.1111/j.1567-1364.2009.00598.x

Hughes Hallett, J. E., Luo, X., & Capaldi, A. P. (2014). State transitions in the TORC1 signaling pathway and information processing in Saccharomyces cerevisiae. *Genetics*, *198*(2), 773–786. https://doi.org/10.1534/genetics.114.168369

Jiang, Y., & Broach, J. R. (1999). Tor proteins and protein phosphatase 2A reciprocally regulate Tap42 in controlling cell growth in yeast. *The EMBO Journal*, *18*(10), 2782–2792. https://doi.org/10.1093/emboj/18.10.2782

Jones, S., Vignais, M. L., & Broach, J. R. (1991). The CDC25 protein of Saccharomyces cerevisiae promotes exchange of guanine nucleotides bound to ras. *Molecular and Cellular Biology*, *11*(5), 2641–2646. https://doi.org/10.1128/mcb.11.5.2641

Kacherovsky, N., Tachibana, C., Amos, E., Fox, D., & Young, E. T. (2008). Promoter binding by the Adr1 transcriptional activator may be regulated by phosphorylation in the DNA-binding region. *PLoS ONE*, *3*(9). https://doi.org/10.1371/journal.pone.0003213

Kataoka, T., Broek, D., & Wigler, M. (1985). DNA sequence and characterization of the S. cerevisiae gene encoding adenylate cyclase. *Cell*, *43*(2 PART 1), 493–505. https://doi.org/10.1016/0092-8674(85)90179-5

Kraakman, L., Lemaire, K., Ma, P., Teunlssen, A. W. R. H., Donaton, M. C. V., Van Dijck, P., Winderickx, J., De Winde, J. H., & Thevelein, J. M. (1999). A Saccharomyces cerevisiae G-protein coupled receptor, Gpr1, is specifically required for glucose activation of the cAMP pathway during the transition to growth on glucose. *Molecular Microbiology*, *32*(5), 1002–1012. https://doi.org/10.1046/j.1365-2958.1999.01413.x

Lempiäinen, H., Uotila, A., Urban, J., Dohnal, I., Ammerer, G., Loewith, R., & Shore, D. (2009). Sfp1 Interaction with TORC1 and Mrs6 Reveals Feedback Regulation on TOR Signaling. *Molecular Cell*, *33*(6), 704–716. https://doi.org/10.1016/j.molcel.2009.01.034

Leverentz, M. K., & Reece, R. J. (2006). Phosphorylation of Zn(II)2Cys6 proteins: A cause or effect of transcriptional activation? *Biochemical Society Transactions*, *34*(5), 794–797. https://doi.org/10.1042/BST0340794

Liu, Z., & Butow, R. A. (1999). A Transcriptional Switch in the Expression of Yeast Tricarboxylic Acid Cycle Genes in Response to a Reduction or Loss of Respiratory Function. *Molecular and Cellular Biology*, *19*(10), 6720–6728. https://doi.org/10.1128/mcb.19.10.6720

Ma, P., Wera, S., Van Dijck, P., & Thevelein, J. M. (1999). The PDE1-encoded low-affinity phosphodiesterase in the yeast Saccharomyces cerevisiae has a specific function in controlling agonist- induced cAMP signaling. *Molecular Biology of the Cell*, *10*(1), 91–104. https://doi.org/10.1091/mbc.10.1.91

MacPherson, S., Larochelle, M., & Turcotte, B. (2006). A Fungal Family of Transcriptional Regulators: the Zinc Cluster Proteins. *Microbiology and Molecular Biology Reviews*, *70*(3), 583–604. https://doi.org/10.1128/mmbr.00015-06

Marion, R. M., Regev, A., Segal, E., Barash, Y., Koller, D., Friedman, N., & O’Shea, E. K. (2004). Sfp1 is a stress- and nutrient-sensitive regulator of ribosomal protein gene expression. *Proceedings of the National Academy of Sciences of the United States of America*, *101*(40), 14315–14322. https://doi.org/10.1073/pnas.0405353101

Martínez-Pastor, M. T., Marchler, G., Schüller, C., Marchler-Bauer, A., Ruis, H., & Estruch, F. (1996). The Saccharomyces cerevisiae zinc finger proteins Msn2p and Msn4p are required for transcriptional induction through the stress response element (STRE). *The EMBO Journal*, *15*(9), 2227–2235. https://doi.org/10.1002/j.1460-2075.1996.tb00576.x

Matsumoto, K., Uno, I., Toh-E, A., Ishikawa, T., & Oshima, Y. (1982). Cyclic AMP may not be involved in catabolite repression in Saccharomyes cerevisiae: evidence from mutants capable of utilizing it as an adenine source. *Journal of Bacteriology*, *150*(1), 277–285. http://www.ncbi.nlm.nih.gov/pubmed/6277865

Nicastro, R., Tripodi, F., Gaggini, M., Castoldi, A., Reghellin, V., Nonnis, S., Tedeschi, G., & Coccetti, P. (2015). Snf1 phosphorylates adenylate cyclase and negatively regulates protein kinase A-dependent transcription in Saccharomyces cerevisiae. *Journal of Biological Chemistry*, *290*(41), 24715–24726. https://doi.org/10.1074/jbc.M115.658005

Nikawa, J., Sass, P., & Wigler, M. (1987). Cloning and characterization of the low-affinity cyclic AMP phosphodiesterase gene of Saccharomyces cerevisiae. *Molecular and Cellular Biology*, *7*(10), 3629–3636. https://doi.org/10.1128/mcb.7.10.3629

Pedruzzi, I., Bürckert, N., Egger, P., & De Virgilio, C. (2000). Saccharomyces cerevisiae Ras/cAMP pathway controls post-diauxic shift element-dependent transcription through the zinc finger protein Gis1. *The EMBO Journal*, *19*(11), 2569–2579. https://doi.org/10.1093/emboj/19.11.2569

Peeters, K., Van Leemputte, F., Fischer, B., Bonini, B. M., Quezada, H., Tsytlonok, M., Haesen, D., Vanthienen, W., Bernardes, N., Gonzalez-Blas, C. B., Janssens, V., Tompa, P., Versées, W., & Thevelein, J. M. (2017). Fructose-1,6-bisphosphate couples glycolytic flux to activation of Ras. *Nature Communications*, *8*(1). https://doi.org/10.1038/s41467-017-01019-z

Peeters, T., Louwet, W., Geladé, R., Nauwelaers, D., Thevelein, J. M., & Versele, M. (2006). Kelch-repeat proteins interacting with the Gα protein Gpa2 bypass adenylate cyclase for direct regulation of protein kinase A in yeast. *Proceedings of the National Academy of Sciences of the United States of America*, *103*(35), 13034–13039. https://doi.org/10.1073/pnas.0509644103

Portela, P., Howell, S., Moreno, S., & Rossi, S. (2002). In vivo and in vitro phosphorylation of two isoforms of yeast pyruvate kinase by protein kinase A. *Journal of Biological Chemistry*, *277*(34), 30477–30487. https://doi.org/10.1074/jbc.M201094200

Reinke, A., Anderson, S., McCaffery, J. M., Yates, J., Aronova, S., Chu, S., Fairclough, S., Iverson, C., Wedaman, K. P., & Powers, T. (2004). TOR Complex 1 Includes a Novel Component, Tco89p (YPL180w), and Cooperates with Ssd1p to Maintain Cellular Integrity in Saccharomyces cerevisiae. *Journal of Biological Chemistry*, *279*(15), 14752–14762. https://doi.org/10.1074/jbc.M313062200

Rittenhouse, J., Moberly, L., & Marcus, F. (1987). Phosphorylation in vivo of yeast (Saccharomyces cerevisiae) fructose-1,6-bisphosphatase at the cyclic AMP-dependent site. *Journal of Biological Chemistry*, *262*(21), 10114–10119.

Robinson, L. C., Gibbs, J. B., Marshall, M. S., Sigal, I. S., & Tatchell, K. (1987). CDC25: A component of the RAS-adenylate cyclase pathway in Saccharomyces cerevisiae. *Science*, *235*(4793), 1218–1221. https://doi.org/10.1126/science.3547648

Rolland, F., De Winde, J. H., Lemaire, K., Boles, E., Thevelein, J. M., & Winderickx, J. (2000). Glucose-induced cAMP signalling in yeast requires both a G-protein coupled receptor system for extracellular glucose detection and a separable hexose kinase-dependent sensing process. *Molecular Microbiology*, *38*(2), 348–358. https://doi.org/10.1046/j.1365-2958.2000.02125.x

Santangelo, G. M. (2006). Glucose Signaling in Saccharomyces cerevisiae. *Microbiology and Molecular Biology Reviews*, *70*(1), 253–282. https://doi.org/10.1128/mmbr.70.1.253-282.2006

Sanz, P., Alms, G. R., Haystead, T. A. J., & Carlson, M. (2000). Regulatory Interactions between the Reg1-Glc7 Protein Phosphatase and the Snf1 Protein Kinase. *Molecular and Cellular Biology*, *20*(4), 1321–1328. https://doi.org/10.1128/mcb.20.4.1321-1328.2000

Sass, P., Field, J., Nikawa, J., Toda, T., & Wigler, M. (1986). Cloning and characterization of the high-affinity cAMP phosphodiesterase of Saccharomyces cerevisiae. *Proceedings of the National Academy of Sciences of the United States of America*, *83*(24), 9303–9307. https://doi.org/10.1073/pnas.83.24.9303

Schepers, W., Van Zeebroeck, G., Pinkse, M., Verhaert, P., & Thevelein, J. M. (2012). In vivo phosphorylation of Ser21 and Ser83 during nutrient-induced activation of the yeast protein kinase A (PKA) target trehalase. *Journal of Biological Chemistry*, *287*(53), 44130–44142. https://doi.org/10.1074/jbc.M112.421503

Schüller, H. J. (2003). Transcriptional control of nonfermentative metabolism in the yeast Saccharomyces cerevisiae. *Current Genetics*, *43*(3), 139–160. https://doi.org/10.1007/s00294-003-0381-8

Smith, J. J., Miller, L. R., Kreisberg, R., Vazquez, L., Wan, Y., & Aitchison, J. D. (2011). Environment-responsive transcription factors bind subtelomeric elements and regulate gene silencing. *Molecular Systems Biology*, *7*, 455. https://doi.org/10.1038/msb.2010.110

Soontorngun, N., Baramee, S., Tangsombatvichit, C., Thepnok, P., Cheevadhanarak, S., Robert, F., & Turcotte, B. (2012). Genome-wide location analysis reveals an important overlap between the targets of the yeast transcriptional regulators Rds2 and Adr1. *Biochemical and Biophysical Research Communications*, *423*(4), 632–637. https://doi.org/10.1016/j.bbrc.2012.05.151

Sutherland, C. M., Hawley, S. A., McCartney, R. R., Leech, A., Stark, M. J. R., Schmidt, M. C., & Hardie, D. G. (2003). Elm1p is one of three upstream kinases for the Saccharomyces cerevisiae SNF1 complex. *Current Biology : CB*, *13*(15), 1299–1305. https://doi.org/10.1016/s0960-9822(03)00459-7

Swinnen, E., Wanke, V., Roosen, J., Smets, B., Dubouloz, F., Pedruzzi, I., Cameroni, E., De Virgilio, C., & Winderickx, J. (2006). Rim15 and the crossroads of nutrient signalling pathways in Saccharomyces cerevisiae. In *Cell Division* (Vol. 1, p. 3). BioMed Central. https://doi.org/10.1186/1747-1028-1-3

Tanaka, K, Matsumoto, K., & Toh-E, A. (1989). IRA1, an inhibitory regulator of the RAS-cyclic AMP pathway in Saccharomyces cerevisiae. *Molecular and Cellular Biology*, *9*(2), 757–768. https://doi.org/10.1128/mcb.9.2.757

Tanaka, K, Nakafuku, M., Tamanoi, F., Kaziro, Y., Matsumoto, K., & Toh-e, A. (1990). IRA2, a second gene of Saccharomyces cerevisiae that encodes a protein with a domain homologous to mammalian ras GTPase-activating protein. *Molecular and Cellular Biology*, *10*(8), 4303–4313. https://doi.org/10.1128/mcb.10.8.4303

Tanaka, Kazuma, Nakafuku, M., Satoh, T., Marshall, M. S., Gibbs, J. B., Matsumoto, K., Kaziro, Y., & Toh-e, A. (1990). S. cerevisiae genes IRA1 and IRA2 encode proteins that may be functionally equivalent to mammalian ras GTPase activating protein. *Cell*, *60*(5), 803–807. https://doi.org/10.1016/0092-8674(90)90094-U

Toda, T, Cameron, S., Sass, P., Zoller, M., Scott, J. D., McMullen, B., Hurwitz, M., Krebs, E. G., & Wigler, M. (1987). Cloning and characterization of BCY1, a locus encoding a regulatory subunit of the cyclic AMP-dependent protein kinase in Saccharomyces cerevisiae. *Molecular and Cellular Biology*, *7*(4), 1371–1377. https://doi.org/10.1128/mcb.7.4.1371

Toda, Takashi, Cameron, S., Sass, P., Zoller, M., & Wigler, M. (1987). Three different genes in S. cerevisiae encode the catalytic subunits of the cAMP-dependent protein kinase. *Cell*, *50*(2), 277–287. https://doi.org/10.1016/0092-8674(87)90223-6

Toda, Takashi, Uno, I., Ishikawa, T., Powers, S., Kataoka, T., Broek, D., Cameron, S., Broach, J., Matsumoto, K., & Wigler, M. (1985). In yeast, RAS proteins are controlling elements of adenylate cyclase. *Cell*, *40*(1), 27–36. https://doi.org/10.1016/0092-8674(85)90305-8

Turcotte, B., Liang, X. B., Robert, F., & Soontorngun, N. (2010). Transcriptional regulation of nonfermentable carbon utilization in budding yeast. In *FEMS Yeast Research* (Vol. 10, Issue 1, pp. 2–13). PMC Canada manuscript submission. https://doi.org/10.1111/j.1567-1364.2009.00555.x

Urban, J., Soulard, A., Huber, A., Lippman, S., Mukhopadhyay, D., Deloche, O., Wanke, V., Anrather, D., Ammerer, G., Riezman, H., Broach, J. R., De Virgilio, C., Hall, M. N., & Loewith, R. (2007). Sch9 Is a Major Target of TORC1 in Saccharomyces cerevisiae. *Molecular Cell*, *26*(5), 663–674. https://doi.org/10.1016/j.molcel.2007.04.020

Wanke, V., Cameroni, E., Uotila, A., Piccolis, M., Urban, J., Loewith, R., & De Virgilio, C. (2008). Caffeine extends yeast lifespan by targeting TORC1. *Molecular Microbiology*, *69*(1), 277–285. https://doi.org/10.1111/j.1365-2958.2008.06292.x

Westholm, J. O., Nordberg, N., Murén, E., Ameur, A., Komorowski, J., & Ronne, H. (2008). Combinatorial control of gene expression by the three yeast repressors Mig1, Mig2 and Mig3. *BMC Genomics*, *9*(SUPPL. 2), 601. https://doi.org/10.1186/1471-2164-9-601

Woods, A., Munday, M. R., Scott, J., Yang, X., Carlson, M., & Carling, D. (1994). Yeast SNF1 is functionally related to mammalian AMP-activated protein kinase and regulates acetyl-CoA carboxylase in vivo. *Journal of Biological Chemistry*, *269*(30), 19509–19515.

Yan, G., Shen, X., & Jiang, Y. (2006). Rapamycin activates Tap42-associated phosphatases by abrogating their association with Tor complex 1. *EMBO Journal*, *25*(15), 3546–3555. https://doi.org/10.1038/sj.emboj.7601239
