# Supplementary material for: A novel yeast hybrid modeling framework integrating Boolean and enzyme-constrained networks enables exploration of the interplay between signaling and metabolism
Source: PLoS Comput Biol. 2021 Apr 9;17(4):e1008891. doi: 10.1371/journal.pcbi.1008891 (PMC8059808; doi:10.1371/journal.pcbi.1008891)
Supplement: S2 Table — (DOCX) [file pcbi.1008891.s008.docx]

S2 Table . Summary of the statistics done comparing the ecModel and the hybrid model in their ability to predict protein abundance.

| Statistics | Respiration | | Fermentation | |
| --- | --- | --- | --- | --- |
|  | ecModel | Hybrid model | ecModel | Hybrid model |
| PCC (statistics) | 0.23379 | 0.18143 | 0.013517 | -0.038213 |
| PCC (P-value) | 0.019 | 0.048 | 0.417 | 0.648 |
| KS (statistics) | 0.50575 | 0.41379 | 0.36667 | 0.0008511 |
| KS (P-value) | 1.7062e-10 | 3.636e-07 | 1.1208e-07 | 0.25 |
| r | 2.62 | 1.56 | 3.56 | 2.33 |
