# Supplementary material for: A novel yeast hybrid modeling framework integrating Boolean and enzyme-constrained networks enables exploration of the interplay between signaling and metabolism
Source: PLoS Comput Biol. 2021 Apr 9;17(4):e1008891. doi: 10.1371/journal.pcbi.1008891 (PMC8059808; doi:10.1371/journal.pcbi.1008891)
Supplement: S1 Fig — Simulations are made with all crosstalk turned on. Panel (A) shows the simulation dynamics going from nutrient-depleted conditions to nutrient-rich conditions and panel (B) shows the simulation dynamics going from nutrient-rich conditions to nutrient depletion. (PDF) [file pcbi.1008891.s003.pdf]

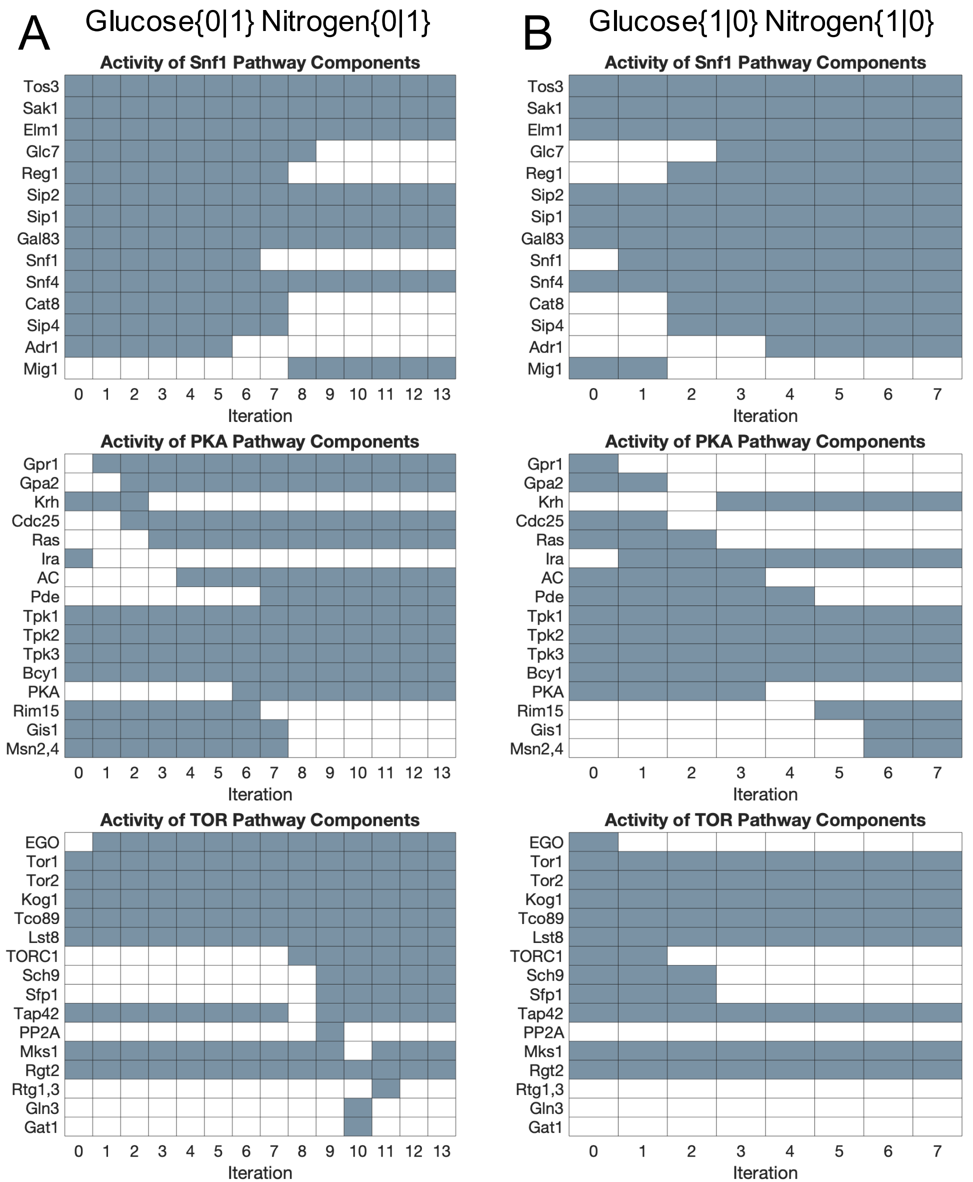
A
Glucose{0|1} Nitrogen{0|1}
B
Glucose{1|0} Nitrogen{1|0}
Activity of Snf1 Pathway Components
Tos3
Sak1
Elm1
Glc7
Reg1
Sip2
Sip1
Gal83
Snf1
Snf4
Cat8
Sip4
Adr1
Mig1
Activity of PKA Pathway Components
Gpr1
Gpa2
Krh
Cdc25
Ras
Ira
AC
Pde
Tpk1
Tpk2
Tpk3
Bcy1
PKA
Rim15
Gis1
Msn2,4
Activity of TOR Pathway Components
EGO
Tor1
Tor2
Kog1
Tco89
Lst8
TORC1
Sch9
Sfp1
Tap42
PP2A
Mks1
Rgt2
Rtg1,3
Gln3
Gat1
Iteration
