# Supplementary material for: A novel yeast hybrid modeling framework integrating Boolean and enzyme-constrained networks enables exploration of the interplay between signaling and metabolism
Source: PLoS Comput Biol. 2021 Apr 9;17(4):e1008891. doi: 10.1371/journal.pcbi.1008891 (PMC8059808; doi:10.1371/journal.pcbi.1008891)
Supplement: S4 Fig — The color of the connectors represents the change in flux from the wild type (WT) hybrid model compared to the SNF1 deletion hybrid model. The FCCs are represented in the model where the WT is compared to the SNF1 deletion case. (PDF) [file pcbi.1008891.s006.pdf]

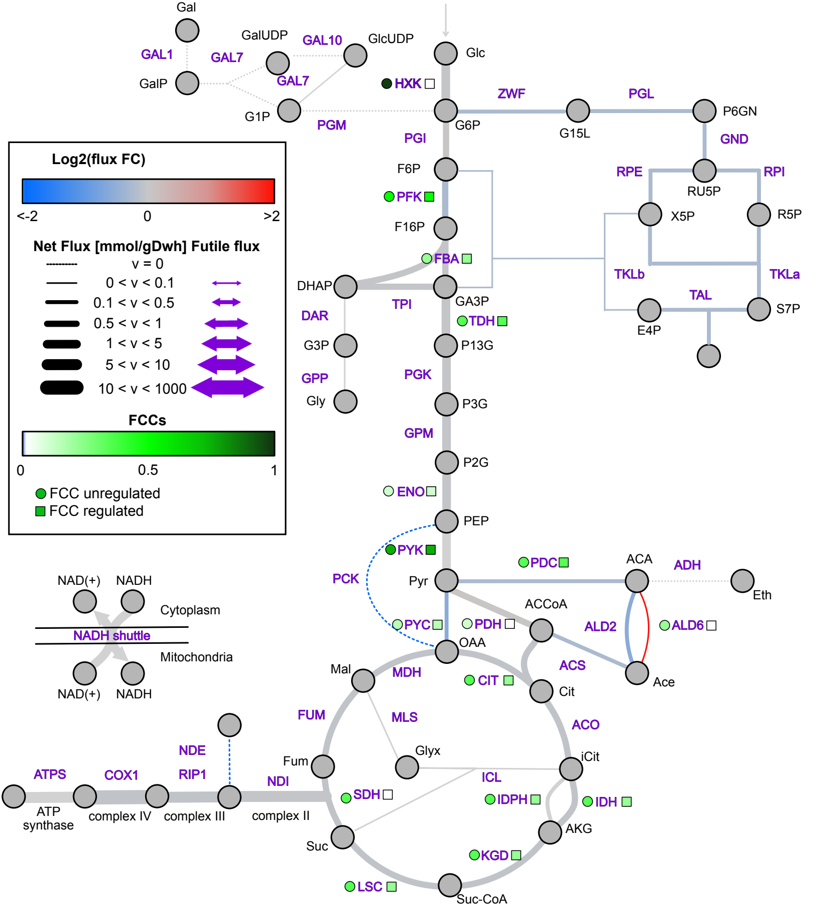

Gal
GAL1
GalP
GAL7
GalUDP
GAL10
GlcUDP
GAL7
G1P
PGM
Glc
HXK
G6P
ZWF
G15L
PGL
P6GN
GND
PGI
F6P
PFK
F16P
FBA
RPE
RU5P
RPI
X5P
R5P
TKLb
TKLa
TAL
E4P
S7P
DHAP
TPI
GA3P
TDH
DAR
G3P
P13G
PGK
GPP
Gly
P3G
GPM
P2G
ENO
PEP
PYK
PCK
Pyr
PDC
ACA
ADH
Eth
ACCoA
PYC
PDH
ALD2
ALD6
OAA
ACS
Ace
Mal
MDH
CIT
Cit
MLS
FUM
ACO
Glyx
iCit
Fum
ICL
SDH
IDPH
IDH
AKG
Suc
KGD
LSC
Suc-CoA
Log2(flux FC)
<-2
0
>2
Net Flux [mmol/gDwh] Futile flux
v = 0
0 < v < 0.1
0.1 < v < 0.5
0.5 < v < 1
1 < v < 5
5 < v < 10
10 < v < 1000
FCCs
0
0.5
1
FCC unregulated
FCC regulated
NAD(+)
NADH
Cytoplasm
NADH shuttle
Mitochondria
NAD(+)
NADH
NDE
ATPS
COX1
RIP1
NDI
ATP synthase
complex IV
complex III
complex II
