# Supplementary material for: A novel yeast hybrid modeling framework integrating Boolean and enzyme-constrained networks enables exploration of the interplay between signaling and metabolism
Source: PLoS Comput Biol. 2021 Apr 9;17(4):e1008891. doi: 10.1371/journal.pcbi.1008891 (PMC8059808; doi:10.1371/journal.pcbi.1008891)
Supplement: Attachment — Submitted filename: Rebuttal.pdf [file pcbi.1008891.s013.pdf]

Reviewer's Responses to Questions

Comments to the Authors:
Please note here if the review is uploaded as an attachment.

Reviewer #1: In this work, the authors present a hybrid yeast model combining signaling, regulation and metabolism. The model offers advantages over purely metabolic models by allowing to analyse the effects of nutrient sensing. The final model is a combination of a signaling and regulatory network for carbon and nitrogen sensing, manually curated by the authors, with a core metabolic model of yeast metabolism obtained by reducing the network of the yeast gecko model. Overall the paper presents a methodological advancement in the combination of boolean-based signaling model with a protein-constrained metabolic model. While the scientific aspect of the manuscript seems solid, and of general interest, for me the main issue is the writing and structure of the manuscript. I found the text not very well written and difficult to follow. I believe the manuscript would benefit from substantial rewriting. Please see further details bellow.

We would like to thank the reviewer for a very careful and thorough evaluation of the manuscript. We have received very much appreciated, constructive and valuable comments and suggestions. We carefully addressed all comments which lead to a revision of the manuscript and the supplementary information.

Major issues:

* In the introduction the reader is only pointed to a diagram that doesn't explain much of the simulation method. The details of simulation are explained at the end, but a brief description (pointing to the methods section for a more detailed description) should be given before explaining the main results.

We have added a brief description of the model integration and simulation algorithm in the introduction section, including a reference to the methods section for further details (Ln139-148).

* Each subsection of the results immediately begins with an enumeration of results. A bit more context into the motivation for each part, and an explanation of why these particular simulations were selected with respect to that motivation, would better help the reader to interpret the results.

We added additional motivation to why we do the particular simulations and also moved information about additional simulations from the supplementary to the main text for the reader to get a more complete picture of the work done.

* I don't understand why are there only 3 figures in the main text and all the others go into supplementary material. Some of these figures are essential for understanding the method and the main findings in the paper.

Thank you for this input. We have decided to make an additional figure (new Figure 3) which summarizes our main results. We have also provided a detailed explanations in the text and

have further cross-references both to supplementary material and to the Method section which we believe will make it equally clear to understand the method and main findings.

* The supplementary file itself is very confusing. There are full paragraphs of text mixed with figures and tables. This file needs to be better organized into supplementary figures, supplementary tables and supplementary text. Also, a lot of the supplementary text contains interpretation of results and discussion. There is no explanation given to the reader at any point on why some of the text is moved into supplementary and what one should expect to find there.

We have now completely revised and reorganized the supplementary material as suggested, we added more cross-references and a better description of the material.

Minor issues:

* Please explain how the thresholds are decided for converting the glucose and nitrogen concentrations into binary values.

This is a very interesting and difficult question in hybrid modelling. The glucose uptake rate value at which a purely respiratory metabolic strategy utilizes the totality of the available pool of enzymes (critical GUR) was obtained from prior simulations of cell physiology at increasing dilution rates with the ecModel. Previous in silico studies (https://doi.org/10.15252/msb.20167411 and https://doi.org/10.1038/srep22264) have shown that, in order to utilize any higher flux of glucose, yeast cells switch their metabolic strategy gradually into a mixed respiro-fermentative metabolism, leveraging both metabolic and protein efficiency. In this study low glucose uptake rates correspond to those values below the critical GUR, and high-levels to those values above it. This is described in the Methods section (line 639). Having a Boolean response to glucose uptake levels is a simplifying assumption of this approach since, in the SNF1 pathway, Mig1 nuclear localisation react to glucose in a gradual manner (https://www.ncbi.nlm.nih.gov/pmc/articles/PMC4007474/). In steady-state, cAMP levels (determining PKA activation) show a linear relationship with glucose. Moreover, the activation of cAMP-PKA follows Weber's law and react to relative changes in glucose more than absolute glucose levels. (https://www.biorxiv.org/content/10.1101/831354v1.full)

* Please specify the growth medium used during simulation.

We have assumed cellular growth on mineral minimal media, supplemented with glucose as a carbon source. As this is a simplified model of central carbon metabolism, the role of all relevant mineral ions in metabolism is not fully described, therefore the media formulation is limited to unbounded uptake of glucose, phosphate, oxygen and water. This is now specified in the methods under the simulations section.

* In lines 231-239 there are multiple p-values presented. Please mention also the statistical test used, sample sizes, effect size, and the respective test statistic.

To calculate this, we used YeastMine with standard settings. YeastMine relies on the use of a hypergeometric distribution for statistical test and the Holm-Bonferroni method for correction

of p-values. We don't use this analysis in any of our major conclusions, but to get a feeling for where the errors are located.

* Fig 3A looks very suspicious, some values reach almost 10^-10, which is probably below the solver precision. Log-changes are a very strange way to compare experimental and simulated fluxes. Why not use the mean squared error (MSE) instead?

The values shown in figure 3A are not a direct output from the solvers used for simulation. These are $\log_{10}$ fold-changes between predicted and experimental individual enzyme demands, therefore the precision of the solver is not an issue for this. Also, previously published studies have been able to generate reliable phenotype predictions relying on enzyme-constrained models even by using default settings of commercial solvers for linear-programming problems (https://doi.org/10.15252/msb.20167411; https://doi.org/10.1038/srep22264; https://doi.org/10.1038/s41467-019-12934-8; 10.1126/scisignal.aaz1482; https://doi.org/10.1038/s41467-019-11581-3; 10.1016/j.isci.2020.101525)

The low values close to 10^{-10} for the $\log_{10}$ fold-changes are obtained when a protein with a predicted usage of zero is present with a non-zero concentration value in the experimental dataset. We substituted all zero values in flux and enzyme usage distributions by 10^{-12}, to avoid arithmetic problems when calculating $\log_{10}$ fold-changes between two distributions. This was done due to the fact that minimum expected values in an enzyme usage distribution are in the order of 10^{-8} mmol/gDw, verified in predicted enzyme usage profiles for the interval 0-0.4h^{-1} of dilution rate. Therefore, the selection of this "detection threshold" in our simulations was based on the characteristic magnitudes of the modelled system while ensuring minimal perturbation of results for analysis purposes.

For quality assessment of predictions of protein demands, we decided to use $\log_{10}$ fold-change between predicted and experimental values, instead of MSE or other error metrics, because this metric can be easily interpreted as the amount of orders of magnitude that predictions deviate from experimental values. In contrast, MSE does not offer a straightforward interpretation of the observed errors; moreover, it is not a dimensionless quantity, which hampers the comparison across conditions due to its dependence on the magnitude of the predicted outcomes.

* Fig 3B: why showing only these 3 proteins when there are more proteomics data ?

We have now included a new figure showing all enzymes.

* Fig 3D: the net flux for PGK and GPM seems higher than futile flux, how is this possible?

Yes, the net flux for PGK is more than 7 times higher than its futile flux; for PGK, the net flux is 15 times higher than the futile one. Actually, this is expected behaviour as futile fluxes are defined as

$$v_i^{futile} = v_i^{forward} - v_i^{net}$$

Therefore, futile fluxes tell us about the amount for forward flux in a reaction that is cancelled out by backward fluxes. Even more, the integration of a regulatory network enables prediction of such futile metabolic fluxes which have extensively been reported to take place within cells for a wide variety of organisms in order to provide them with robustness to environmental perturbations (https://www.biorxiv.org/content/10.1101/2020.03.02.973669v1.abstract, https://www.nature.com/articles/ncomms11933#Sec8, https://www.nature.com/articles/nchembio.2077, https://www.nature.com/articles/s41589-019-0364-9).

Reviewer #2: This paper aims do provide a novel hybrid modelling framework to integrate (nutrient-induced) signalling, regulation and metabolism, which is applied to yeast and used to perform some simulations.
The overall idea for the framework is well designed and the goal is worthy of research, being a major aim of systems biology. However, I believe that the work has several limitations, mainly regarding the results presented, which do not seem to justify the authors' claims.

We would like to thank the reviewer for a very careful and thorough evaluation of the manuscript. We have received very much appreciated, constructive and valuable comments and suggestions. We carefully addressed all comments which lead to a revision of the manuscript and the supplementary information.

Firstly, regarding the model, it should be clear from the beginning this not a genome-scale model, even in the metabolic part and that the signalling and regulatory layers are very limited. For instance, this is not clear in the abstract of the work.

We have now revised the Abstract, indicating that the enzyme-constrained metabolic model is limited to the central carbon metabolism and that the signalling layer refers to the main pathways of glucose and nitrogen signalling. We have also revised the Introduction section where this is further explained (Ln134-148)

Also, the reported simulations are very limited and provide results that are not convincing. The errors provided for the proteomics datasets are all in the high orders of magnitude (2 to 4); while improvements are visible the obtained errors are still around 100 fold. Also, the reported use of iso-enzymes is only somewhat visible in fermentation.

Enzyme usages predicted by our hybrid model (interpreted as enzyme demands for metabolic reactions) are compared to absolute concentrations of proteins in biological samples. Absolute quantification of proteins is still an open and challenging issue, the current state of the art shows that label-free methods, such as iBAQ combined with the use of external standards, yield measurements that differ even in orders of magnitude for proteins of known concentration. This is clearly shown by any calibration curve for label-free proteomics methods based on the use of UPS2 standard (https://doi.org/10.1002/pmic.201300135, https://doi.org/10.1016/j.jprot.2012.06.020).

Moreover, when comparing our enzyme usage predictions to proteomics data, it turns out that for the respiratory condition, **40.83%** of the proteins in the model are predicted in the same order magnitude as their experimental values, 31.66% are predicted with an error between

one and two orders of magnitude, and 27.5% with errors higher than two orders of magnitude, showing a median log_{10} fold-change of 1.19. For the fermentative condition, **65.51%** of the proteins are predicted within the same order of magnitude as their experimental measurements, showing a median log_{10} fold-change of 0.68. Altogether, this shows that our hybrid model is able to predict protein abundances with an accuracy that is comparable to the one of the current absolute protein quantification methods (https://www.biorxiv.org/content/10.1101/2020.03.23.998237v1), which, to our knowledge, outperforms all of the currently available constraint-based methods for protein allocation prediction.

To clarify the improvement in the usage of isoenzymes we added a figure visualizing the effects for both fermentation and respiration (new Figure 3).

Finally, the Crabtree effect reported was already reported for GECKO models alone, so it is not clear where the improvement is. The relationship to ageing is also not convincing and its significance not well explained.

We were probably less careful when presenting this result. In the original submission in the Abstract we state: *The model was capable of reproducing the regulatory effects that are associated with the Crabtree effect and glucose repression.* Where we should emphasis the 'regulatory effects associated with..', so we do not claim that we are predicting the Crabtree effect with our hybrid model, as the ecModel layer is able to do it by itself, therefore the good agreement of exchange fluxes predictions with experimental data around the critical dilution rate serves as a validation for this model layer. By comparing the ecModel and the hybrid model we can see that the signalling layer changes the metabolism in the same way that is expected after the onset of the long term Crabtree effect, also serving as a good validation benchmark for our hybrid approach. The major improvements in terms of predictive performance are:

1) Prediction of protein demands for both respiratory and mixed metabolism outperforms those of any other constraint-based method for protein allocation at the level of individual enzymes.
2) Our hybrid model is able to capture diverse utilization of isoenzymes for metabolic reactions, as suggested by proteomics data, even at protein-limited conditions. This is not possible for any method that relies just on optimality principles; therefore the incorporation of a regulatory layer provides a biological explanation to the deviations from optimality suggested by omics datasets.

In aging, caloric restriction has been long associated with prolonged chronological lifespan. This phenomenon is known to work through SNF1 where Snf1 activation is necessary for CR related prolongation of chronological lifespan. With our hybrid model, we could, in addition to the novel NDI/NDE connection to Snf1, also predict and attribute known connections to Snf1 regulation and chronological lifespan. The expression of ACS1 during CR is attenuated in a Snf1 deletion which is also what we see in our model that this expression would be active during respiration is nothing we see in the pure ecModel however with our hybrid model we can predict its usage and also by simulating mutants we can attribute it to the relevant pathway (https://www.ncbi.nlm.nih.gov/pmc/articles/PMC5472825/). We also, now compare regulatory effects of the hybrid model on genes known to be regulated in chronologically

long-lived cells by a different mechanism and can further strengthen the connection of Snf1 and caloric restriction.

So, although I value the work on the framework and the thorough work on the signalling part of the model, the overall result does not seem to justify the publication.
There are also some language problems; the overall paper should be read, as there are a few typos (e.g. investigat - 28; singaling - 39, trough - 548) and in many cases sentences are confusing missing commas to make them more readable (as an example consider the sentence in rows 89-90). The language in S1 text is even less carefully written (consider one example of a sentence: "As can be seen by the summery statistics, prediction of individual proteins are difficult. ").

We have extensively revised both the main text and the SI (both when it comes to language and to the clarity of the text).

Have all data underlying the figures and results presented in the manuscript been provided?
Large-scale datasets should be made available via a public repository as described in the *PLOS Computational Biology* data availability policy, and numerical data that underlies graphs or summary statistics should be provided in spreadsheet form as supporting information.

Reviewer #1: None

Reviewer #2: Yes
